# Circadian clocks guide dendritic cells into skin lymphatics

**DOI:** 10.1038/s41590-021-01040-x

**Published:** 2021-10-18

**Authors:** Stephan J. Holtkamp, Louise M. Ince, Coline Barnoud, Madeleine T. Schmitt, Flore Sinturel, Violetta Pilorz, Robert Pick, Stéphane Jemelin, Michael Mühlstädt, Wolf-Henning Boehncke, Jasmin Weber, David Laubender, Julia Philippou-Massier, Chien-Sin Chen, Leonie Holtermann, Dietmar Vestweber, Markus Sperandio, Barbara U. Schraml, Cornelia Halin, Charna Dibner, Henrik Oster, Jörg Renkawitz, Christoph Scheiermann

**Affiliations:** 1 Biomedical Center (BMC), Institute for Cardiovascular Physiology and Pathophysiology, Walter Brendel Center for Experimental Medicine (WBex), Faculty of Medicine, Ludwig-Maximilians-Universität Munich, Planegg-Martinsried, Germany; 2 Department of Pathology and Immunology, Faculty of Medicine, University of Geneva, Geneva, Switzerland; 3 Laboratory ‘Cell Biology of the Immune System’, Biomedical Center (BMC), Institute for Cardiovascular Physiology and Pathophysiology, Walter Brendel Center for Experimental Medicine (WBex), Faculty of Medicine, Ludwig-Maximilians-Universität Munich, Planegg-Martinsried, Germany; 4 Department of Medicine, Division of Endocrinology, Diabetes, Nutrition and Patient Education, University Hospitals of Geneva, Geneva, Switzerland; 5 Department of Cell Physiology and Metabolism, University of Geneva, Geneva, Switzerland; 6 Diabetes Center, Faculty of Medicine, University of Geneva, Geneva, Switzerland; 7 Institute of Genetics and Genomics of Geneva (iGE3), University of Geneva, Geneva, Switzerland; 8 Institute of Neurobiology, University of Lübeck, Lübeck, Germany; 9 Division of Dermatology and Venereology, Department of Medicine, University Hospitals of Geneva, Geneva, Switzerland; 10 Max Planck Institute of Neurobiology, Martinsried, Germany; 11 Laboratory for Functional Genome Analysis, Gene Center Munich, Ludwig-Maximilians-Universität Munich, Munich, Germany; 12 Department of Vascular Cell Biology, Max Planck Institute for Molecular Biomedicine, Münster, Germany; 13 Institute of Pharmaceutical Sciences, ETH Zurich, Zurich, Switzerland

**Keywords:** Adaptive immunity, Lymphatic vessels

## Abstract

Migration of leukocytes from the skin to lymph nodes (LNs) via afferent lymphatic vessels (LVs) is pivotal for adaptive immune responses^1,2^. Circadian rhythms have emerged as important regulators of leukocyte trafficking to LNs via the blood^3,4^. Here, we demonstrate that dendritic cells (DCs) have a circadian migration pattern into LVs, which peaks during the rest phase in mice. This migration pattern is determined by rhythmic gradients in the expression of the chemokine CCL21 and of adhesion molecules in both mice and humans. Chronopharmacological targeting of the involved factors abrogates circadian migration of DCs. We identify cell-intrinsic circadian oscillations in skin lymphatic endothelial cells (LECs) and DCs that cogovern these rhythms, as their genetic disruption in either cell type ablates circadian trafficking. These observations indicate that circadian clocks control the infiltration of DCs into skin lymphatics, a process that is essential for many adaptive immune responses and relevant for vaccination and immunotherapies.

## Abstract (web summary)

Scheiermann and colleagues show that circadian clocks control the infiltration of dendritic cells into skin lymphatics in mice and humans, with a peak migration to the lymph nodes during the rest phase.

## Main

Steady-state migration of dermal DCs into afferent LVs is tightly regulated by a variety of promigratory factors, including the CCL21–CCR7 chemokine axis and the adhesion molecules LYVE-1, CD99 and JAM-A^1,2,5–12^. In the LN, ~24-h-long circadian rhythms influence the homing capacity and function of lymphocytes^4,13–16^; however, whether the draining of leukocytes from tissues occurs in a rhythmic manner is unknown.

**Fig. 1:**
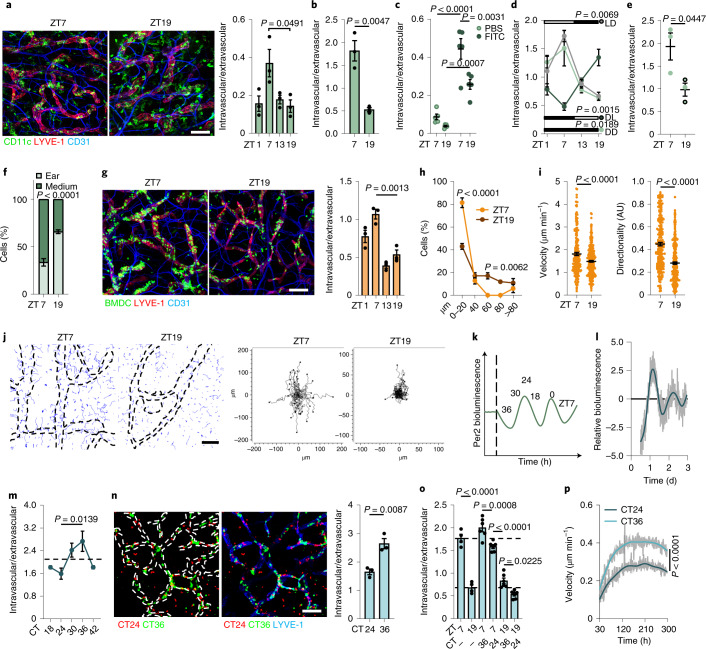
Migration of dermal DCs into skin lymphatics is circadian. **a**,**b**, Crawl-in assays of ear CD11c^+^ cells in LVs after 6 h (**a**) or 24 h (**b**); *n* = 3 mice, one-way analysis of variance (ANOVA) with Tukey’s post test (**a**) and unpaired Student’s *t*-test (**b**). **c**, Ear CD11c^+^ DCs in LVs after 24-h FITC painting; *n* = 5 mice from two independent experiments; two-way ANOVA with Sidak post test. **d**, Ear CD11c^+^ cells in LVs in light:dark (LD), dark:light (DL) or constant darkness (DD) crawl-in assays; *n* = 3 mice; one-way ANOVA. **e**, CD11c^+^langerin^–^ DCs after ear crawl-in assays; *n* = 3 mice; unpaired Student’s *t*-test. **f**, Flow cytometry of ear and medium CD11c^+^MHCII^+^CD103^–^EpCAM^–^ cDC2s after crawl-out assays; *n* = 3 mice; two-way ANOVA. **g**, Localization of ear bone-marrow-derived DCs (BMDCs) after 3-h crawl-in assays; *n* = 3 mice; one-way ANOVA with Tukey’s post test. **h**, Localization of ear BMDCs after 1-h crawl-in assays; *n* = 4 mice; unpaired Student’s *t*-test. **i**, Velocity and directionality of BMDCs in ear crawl-in assays; *n* = 217 and 301 cells, respectively, from four mice from four independent experiments; unpaired Student’s *t*-test; AU, arbitrary units. **j**, Migration tracks (blue, left) of BMDCs overlaid onto LVs (dotted lines, left) and coordinates (right); *n* = 34 cells from four mice from four independent experiments. **k**, BMDC synchronization with serum at different circadian times (CTs). **l**, Bioluminescence oscillations from lipopolysaccharide (LPS)-activated *Per2*:*Luc* BMDCs after synchronization with serum. **m**, Synchronized BMDCs after 3-h ear crawl-in assays (non-synchronized control is represented by dotted lines). CT18/CT42 is double plotted to facilitate viewing; *n* = 4 mice; one-way ANOVA with Tukey’s post test. **n**, Localization of CT24 and CT36 synchronized BMDCs after crawl-in assays; *n* = 4 mice; unpaired Student’s *t*-test. **o**, Localization of CT24 and CT36 synchronized BMDCs after crawl-in assays; *n* = 4 non-synchronized and *n* = 6 synchronized BMDCs; one-way ANOVA with Tukey’s post test. **p**, Velocity of synchronized BMDCs in collagen migration assays; *n* = 3 mice; Kruskal–Wallis test with Dunn’s post test. Scale bars, 100 µm. Data are representative of at least two independent experiments (**a**, **b**, **d**–**h**, **m**–**p**). All data are represented as mean ± s.e.m. Source data

**Extended Data Fig. 1:**
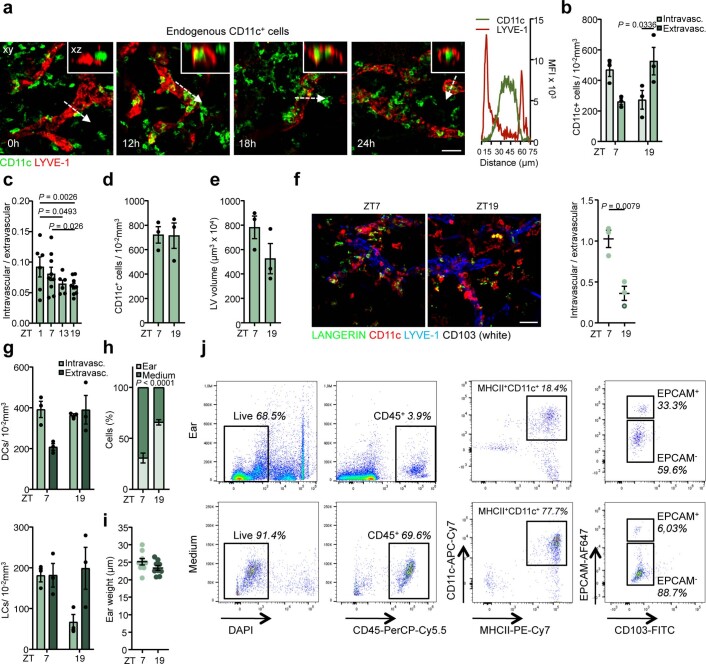
Endogenous CD11c^+^ cell analysis. **a**–**d**, Endogenous crawl-in assay with increasing incubation times, intraluminal CD11c^+^ cells can be identified in LYVE-1^+^ lymphatic vessels (LVs) by fluorescence line plot analysis. Scale bar, 50 µm and 10 µm for orthogonal views, **a**, representative of 2 independent experiments. **b**, Representative numbers of CD11c^+^ cells within or outside of LVs after a 24 h endogenous crawl-in assay, normalized to LV volume and used to calculate the intravascular versus extravascular ratio, n = 3 mice, data are representative of 5 independent experiments, two-way ANOVA with Šidák correction. **c**, Ratio of intravascular versus extravascular CD11c^+^ cells, directly after harvest, n = 6, 9,6, 9 mice, data are representative of 3 independent experiments; one-way ANOVA with Tukey’s post-test. **d**, Total number of CD11c^+^ cells counted per field of view after a 24 h endogenous crawl-in assay, n = 3 mice, data are representative of 5 independent experiments. **e**, Analysed LV volume per field of view, n = 3 mice, data are representative of 5 independent experiments. **f**, 24 h endogenous assay staining for LANGERIN, CD11c and LYVE-1 to measure the intravascular versus extravascular ratio of Langerhans cells (LCs), unpaired student’s *t*-test, scale bar, 50 µm, n = 3 mice, data and images are representative of 2 independent experiments. **g**, Representative numbers of DCs and LCs within or outside of LVs after a 24 h endogenous crawl-in assay, n = 3 mice, data are representative of 2 independent experiments. **h**, Relative numbers of emigrated (medium) or resident (ear) MHCII^+^ CD11c^+^ EPCAM^+^ LCs after a 24 h crawl-out assay, measured by flow cytometry, n = 3 mice, data are representative of 3 independent experiments, two-way ANOVA with Šidák correction. **i**, Weight of ears used for crawl out assays, n = 9 mice from 3 independent experiments, two-way ANOVA. **j**, Gating strategy of EPCAM^+/-^ CD11c^+^ MHCII^+^ dendritic cells (DCs) resident in ears or emigrated into medium. All data represented as mean ± s.e.m. Source data

To address this question, we collected mouse ear skin at different times of the day (that is, zeitgeber time 1 (ZT1; 1 h after light onset in a 12-h light/12-h dark environment; ‘morning’), ZT7 (‘day’), ZT13 (‘evening’) and ZT19 (‘night’)) and cultured the explants for 6 h in medium. We then quantified the location of tissue-endogenous CD11c^+^ DCs inside LYVE-1^+^ skin lymphatics by immunofluorescence imaging (Fig. 1a and Extended Data Fig. 1a,b). Infiltration of CD11c^+^ cells into the LVs peaked at ZT7 (day) and troughed at ZT19 (night) (Fig. 1a and Supplementary Table 1). Additional quantification of the location of CD11c^+^ DCs in the ear at steady state at ZT1, ZT7, ZT13 and ZT19 without ensuing culture confirmed a stronger intralymphatic presence of cells during the day than at night (Extended Data Fig. 1c). Explants that were collected at ZT7 and ZT19 and cultured for 24 h still exhibited higher CD11c^+^ infiltration in LVs at ZT7 than at ZT19 (Fig. 1b and Extended Data Fig. 1b), indicating that night migration did not catch up to the day migration. The diurnal migration of CD11c^+^ DCs into LVs was also detected after the topical application of fluorescein isothiocyanate (FITC), an inflammatory stimulus (Fig. 1c), indicating that the migration differences were maintained during inflammation. The amount of cells analyzed and the overall LV volume was similar at all time points (Extended Data Fig. 1d,e).

Circadian rhythms are defined by their persistence in the absence of external entraining factors, such as rhythmic light onset and offset. To investigate whether the oscillations in DC migration into LVs were circadian in nature, mice were housed in constant darkness. The migration differences of CD11c^+^ DCs in LVs between the peak at ZT7 (day) and the trough at ZT19 (night) continued in conditions of constant darkness, demonstrating these oscillations to be bona fide circadian (Fig. 1d). The oscillations adjusted to a 12-h inverted light–dark cycle (Fig. 1d), indicating that they could be phase shifted and entrained by light, an additional feature of circadian rhythms. This indicated that CD11c^+^ DC migration into skin LVs was driven by endogenous circadian rhythms and did not represent solely a response to a rhythmic day–night environment.

In situ whole-mount staining of ears identified that CD11c^+^langerin^–^CD103^–^ dermal conventional DCs (cDC2s) and CD11c^+^langerin^+^ Langerhans cells (LCs) preferentially migrated into LVs at ZT7 (day) compared to at ZT19 (night), while very few CD11c^+^langerin^–^CD103^+^ cDC1s were detected (Fig. 1e and Extended Data Fig. 1f,g). Flow cytometry of the CD11c^+^MHCII^hi^ cells that emigrated from the skin to the culture medium at ZT7 and ZT19 (Fig. 1f and Extended Data Fig. 1h–j) further indicated that dermal CD103^–^EpCAM^–^ DCs and CD103^–^EpCAM^+^ LCs emigrated from the skin in a highly time-of-day-dependent manner, peaking at ZT7 (day).

**Extended Data Fig. 2:**
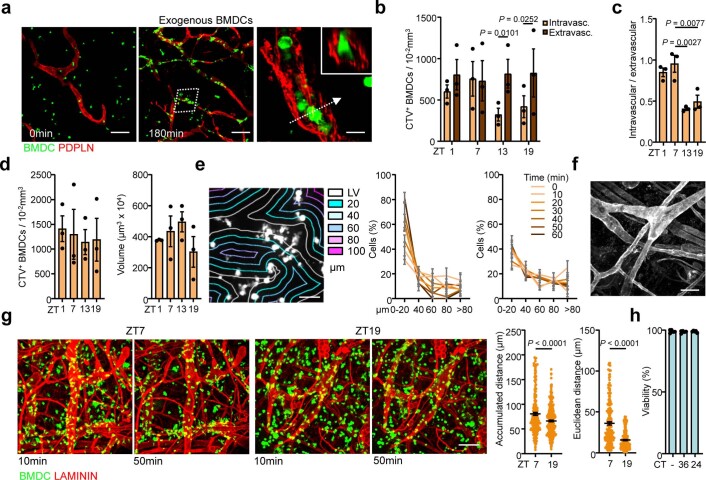
Exogenous BMDC analysis. **a**, **b**, BMDCs of a 3 h exogenous crawl visualized inside the PODOPLANIN^+^ LV lumen using orthogonal views. Scale bar, 70 µm and 10 µm for orthogonal views, **a**. **b**, Representative numbers of BMDCs within or outside of LYVE-1^+^ LVs normalized to vessel volume after a 3 h exogenous crawl in assay, n = 3 mice, data are representative of 5 independent experiments. **c**, Intravascular versus extravascular ratio of individual BMDC batches after a 3 h incubation time. **d**, Total count of BMDCs per field of view normalized to LV volume (left) and calculated LV volume (right), one-way ANOVA with Tukey’s multiple comparisons test, n = 3 mice from 4 independent experiments. **e**, BMDC crawl-in assays with increasing incubation times and analysed by distance dependent zone segmentation of the lymphatic interstitial area, scale bar, 50 µm. Relative accumulation of BMDCs after 0–60 min incubation shown for *Zeitgeber* Time (ZT) 7 and 19, n = 4 mice, data are representative of 2 independent experiments. **f**, Representative whole mount staining from 2 independent experiments of the skin for LAMININ used for live cell imaging, scale bar, 50 µm. **g**, Live cell imaging of BMDCs administered to split ears harvested at ZT7 or ZT19 for 50 min and quantification of accumulated and euclidean distance, unpaired student’s *t*-test, 220 BMDCs for ZT7, 300 BMDCs for ZT19 from 4 mice from 4 independent experiments, scale bar, 100 µm. **h**, Viability of control and circadian time (CT) 36 / CT24 BMDCs after serum shock synchronization measured by flow-cytometry, n = 3 donor mice, data are representative from 3 independent experiments. All data are represented as mean ± s.e.m. Source data

To tease apart the role of the microenvironment from cell-autonomous processes, batches of LPS-activated BMDCs^17^ were generated in vitro. Circadian clocks are desynchronized in culture, and thus BMDCs have no circadian rhythm at the population level^18^. BMDCs were added for 3 h onto split ears collected at ZT1, ZT7, ZT13 and ZT19 from mice housed in light cabinets exhibiting lighting regimens timed for a 6-h difference from each other, enabling the simultaneous collection of ears at four different times of the day (Extended Data Fig. 2a). Using quantitative immunofluorescence imaging assays, we found elevated numbers of labeled leukocytes in the lymphatic capillaries of ZT7 (day) ears compared to ZT19 (night) ears (Fig. 1g and Extended Data Fig. 2b,c). The number of transferred BMDCs present in the skin explants and the LV volume did not differ between time points (Extended Data Fig. 2d). When the BMDC–skin explant culture time (migration time) was shortened to 10–60 min, we detected the accumulation of BMDCs in close proximity to LVs only in the ZT7 (day) ears (Fig.1h and Extended Data Fig. 2e), indicating that diurnal migration differences occur early on. In addition, live imaging of the skin explants showed that BMDCs added onto ZT7 (day) ears exhibited higher velocity and directionality as well as increased total and Euclidean distance compared to BMDCs added to ZT19 (night) ears (Fig. 1i,j, Extended Data Fig. 2f,g and Supplementary Videos 1 and 2). These observations indicated that the ear microenvironment governed the migration capacity of BMDCs.

To assess the influence of circadian rhythms in DCs, LPS-activated BMDCs derived from mice expressing a fusion protein of the circadian protein PER2 and luciferase (PER2–Luc)^19^ were treated with medium containing 50% serum (‘serum shock’) (Fig. 1k)^18,20–22^, which synchronized PER2–Luc expression (Fig. 1l) without affecting cell viability (Extended Data Fig. 2h). When BMDCs collected 24 h and 36 h after synchronization (CT24 and CT36, respectively) were added onto split ears from ZT7 mice, PER2–Luc^lo^ CT36 BMDCs (day BMDCs) migrated more efficiently into the afferent lymphatics of skin explants than PER2–Luc^hi^ CT24 BMDCs (night BMDCs) (Fig. 1m). The same results were observed when PER2–Luc^lo^ CT36 synchronized and PER2–Luc^hi^ CT24 synchronized BMDCs were added together onto the same ZT7 ears (Fig. 1n and Supplementary Video 3), indicating that rhythmicity of migration into LVs was due to a DC-intrinsic mechanism. Using day (PER2–Luc^lo^ CT36) BMDCs on day ears (ZT7) showed maximal cell infiltration into LVs, while night (PER2–Luc^hi^ CT24) BMDCs on night ears (ZT19) showed minimal cell infiltration (Fig. 1o). Day (PER2–Luc^lo^ CT36) BMDCs on night (ZT19) ears as well as night (PER2–Luc^hi^ CT24) BMDCs on day ears (ZT7) resulted in an intermediate capacity of BMDCs to immigrate into LVs (Fig. 1o), demonstrating that in-phase synchronization of both cells and environment provided maximal and minimal migration capacities, respectively. Synchronized PER2–Luc^lo^ CT36 (the time of maximal day migration capacity) BMDCs migrated better than unsynchronized BMDCs (Fig. 1m,o), indicating a benefit of rhythms for overall DC migration behavior. In addition, PER2–Luc^lo^ CT36 day BMDCs migrated with a higher velocity than PER2–Luc^hi^ CT24 night BMDCs in a collagen gel chemotaxis assay (Fig. 1p). These observations indicate that dermal DC migration into afferent lymphatics is controlled by both microenvironmental and DC-autonomous mechanisms.

**Fig. 2:**
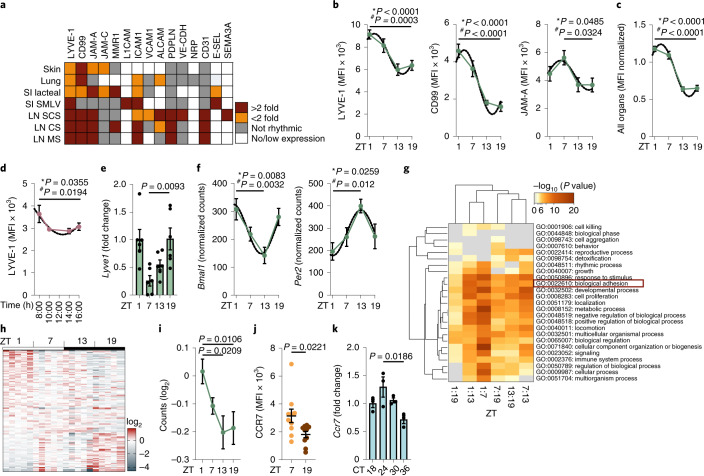
Diurnal expression of promigratory factors in LECs and DCs. **a**, Immunofluorescence screen of molecules expressed in LYVE-1^+^ ear LVs. No/low expression is <1.5% of max mean fluorescence intensity (MFI). Each square represents *n* = 5 mice with four ZTs measured. SI, small intestine;SMLV, sub-mucosal lymphatic vessel;SCS, subcapsular sinus;CS, cortical sinus;MS, medullary sinus. **b**, Expression profiles of molecules in LYVE-1^+^ ear LVs. **c**, Integration of all rhythmic expression profiles across organs collapsed into one graph; *n* = 5 mice from two independent experiments. **d**, Expression profile of LYVE-1 in LVs in human skin biopsies from *n* = 5–9 individuals; asterisks show results from one-way ANOVA, and number signs show results from cosinor analysis (**b**–**d**). **e**–**i**, RNA analyses of dermal LECs sorted at four different ZTs. **e**, Normalized dermal LEC *Lyve1* expression; *n* = 3 mice representative of two independent experiments; one-way ANOVA with Tukey’s post test. **f**, Normalized clock gene counts determined from RNA sequencing of sorted dermal LECs; *n* = 5 mice; asterisks show results from one-way ANOVA, and number signs show results from cosinor analysis. **g**, Significant Gene Ontology (GO) cluster enrichment between different ZTs of dermal LECs. **h**, Heat map of significantly rhythmically expressed adhesion genes (GO:0022610) in dermal LECs. **i**, Integration of all genes from **h** collapsed into one graph; *n* = 82 genes; one-way ANOVA with Tukey’s post test. **j**, Flow cytometry of CCR7 on CD11c^+^MHCII^+^CD103^–^EpCAM^–^ cDC2s; *n* = 10 mice from two independent experiments; unpaired Student’s *t*-test. **k**, Relative *Ccr7* expression in synchronized BMDCs across four CTs and normalized to CT18; *n* = 3 mice representative of two independent experiments; one-way ANOVA with Tukey’s post test. Dotted lines represent fit cosinor curves. All data are represented as mean ± s.e.m. Source data

**Extended Data Fig. 3:**
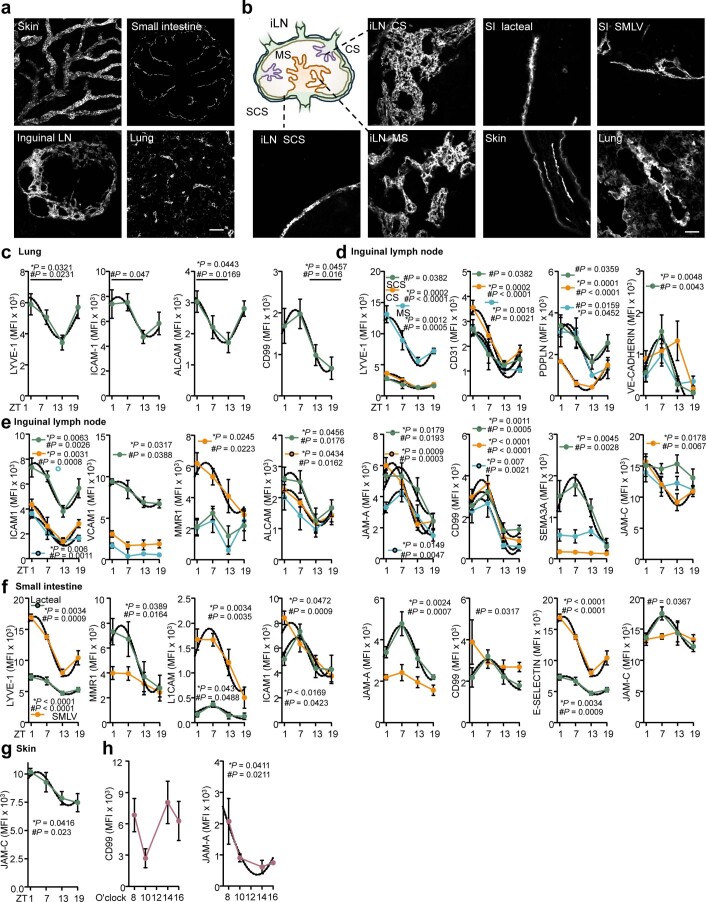
LEC protein analysis. **a**, **b**, LYVE-1^+^ staining overviews of skin, small intestine (SI), inguinal lymph node (iLN) and lung tissues used for quantitative analysis. Scale bar, 150 µm, 100 µm for skin, representative images of 2 independent experiments, **a**. **b**, Respective sub-structures and higher magnifications for analysis. Schematic view of the iLN marks lymphatic sinusoids. CS = cortical sinus, MS = medullary sinus, SCS = subcortical sinus, SMLV = sub-mucosal LV, representative images of 2 independent experiments. Scale bar, 50 µm. **c**–**g**, Quantitative immuno-fluorescence microscopy screen of proteins on LVs in mouse lung, **c**, iLN, **d**, **e**, SI, **f**, and skin, **g**, sections as well as sections from human skin biopsies, **h**. * = one-way ANOVA, # = cosinor analysis, n = 5 for murine (data are representative of 2 independent experiments) and 7,12,7,5 (CD99) and 5,8,4,7 (JAM-A) for human samples. Dotted line represents fit curves. All data are represented as mean ± s.e.m. Source data

We next used quantitative in situ imaging approaches to assess whether the signals involved in DC trafficking to the LVs oscillated in LECs (Extended Data Fig. 3a,b). Afferent LECs in epithelial barrier organs, such as skin, lung and gut but also in LNs, had a tissue-specific temporal expression profile of adhesion molecules (Fig. 2a, Extended Data Fig. 3c–g and Supplementary Table 2). In the skin, we observed strong oscillations of LYVE-1, CD99, JAM-A and JAM-C, molecules that have been implicated in steady-state DC migration^23^, while other adhesion molecules were present but not oscillatory (such as VE-cadherin and PECAM-1) or not expressed at all (such as VCAM-1 and ICAM-1; Fig. 2a,b and Extended Data Fig. 3g). Integrating the profiles from all signals across all organs over time revealed a peak in the expression of oscillatory signals during the day (Fig. 2c), indicating that afferent LECs in the assessed organs have a higher leukocyte recruitment capacity at this time. Of relevance, we detected an analogous rhythm in the expression patterns of LYVE-1 and JAM-A in human skin biopsies (Fig. 2d and Extended Data Fig. 3h). In contrast to murine skin, human dermal LVs showed a nadir in the expression of migratory signals around 12:00 (Fig. 2d and Extended Data Fig. 3h), suggesting that in mice and humans, these signals exhibit a trough during the active phase of these organisms.

**Extended Data Fig. 4:**
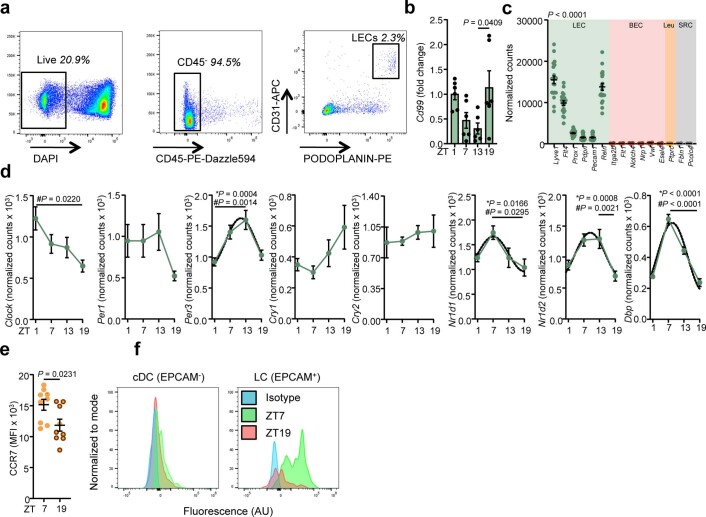
LEC mRNA and DC protein analysis. **a**, Gating strategy of sorted dermal CD31^+^ PODOPLANIN^+^ lymphatic endothelial cells (LECs) for qPCR and RNA-sequencing analyses. **b**, Relative mRNA expression of *Cd99* in sorted dermal LECs after qPCR analysis, one-way ANOVA with Tukey’s post-test, n = 3 mice, data are representative of 2 independent experiments. **c**, Expression of LEC, blood endothelial cell (BEC), leukocyte (leu) and stromal cell (SRC)-specific genes in sorted dermal LECs presented as normalized counts and analysed by RNA sequencing, one-way ANOVA, n = 20. **d**, Clock gene expression of sorted dermal LECs across 4 time points measured and depicted as normalized counts. * = one-way ANOVA, # = cosinor analysis, n = 5 mice. **e**, **f**, Flow-cytometric analysis of CCR7 on emigrated DCs after a 24 h crawl-out assay. **e**, MFI of CCR7 on Langerhans cells (LCs), n = 9 mice from 2 independent experiments. **f**, Histograms of CCR7 MFI on emigrated cDCs (EPCAM^-^) and LCs (EPCAM^+^) in comparison to an isotype control, unpaired student’s *t*-test. All data are represented as mean ± s.e.m. Source data

Quantitative real-time PCR (qPCR) analyses of sorted mouse dermal CD31^+^podoplanin^+^ LECs at ZT1, ZT7, ZT13 and ZT19 (Extended Data Fig. 4a) revealed a night peak in the mRNA amounts for *Lyve1* and *Cd99* (Fig. 2e and Extended Data Fig. 4b), indicating that these rhythms are under transcriptional control. To obtain unbiased insights into the global time-of-day-dependent changes within LECs, we performed bulk RNA sequencing analyses of sorted dermal CD31^+^podoplanin^+^ LECs at ZT1, ZT7, ZT13 and ZT19 (Extended Data Fig. 4c). LECs exhibited strong oscillations in core components of the circadian clock, including the transcription factors *Bmal1* and *Per2* (Fig. 2f and Extended Data Fig. 4d). Moreover, GO enrichment analyses yielded a highly rhythmic cellular profile, particularly for adhesion processes (GO:0022610) (Fig. 2g–i and Supplementary Table 3). Flow cytometry on the culture medium of ear explants at ZT7 (day) and ZT19 (night) indicated that CD103^–^EpCAM^–^ dermal cDCs and CD103^–^EpCAM^+^ LCs exhibited elevated expression of CCR7 at ZT7 compared to ZT19 (Fig. 2j and Extended Data Fig. 4e,f), while *Ccr7* mRNA in synchronized CT18, CT24, CT30 and CT36 BMDCs showed a circadian expression profile, peaking at CT24 (Fig. 2k). These observations indicated that LECs express a circadian clock machinery, and LECs and DCs express promigratory factors in a rhythmic fashion.

**Fig. 3:**
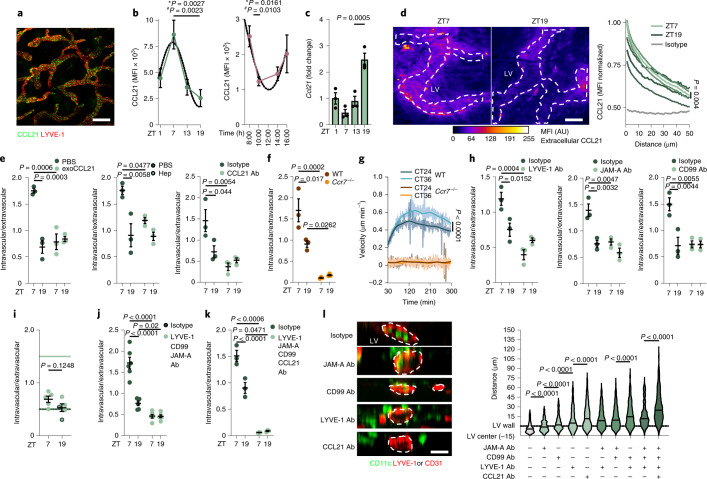
Chronopharmacology of circadian DC migration. **a**, Imaging of CCL21 in permeabilized ears. Image is representative of two independent experiments. Scale bar, 70 µm. **b**, Quantitative immunofluorescence screen of CCL21 in LYVE-1^+^ LVs in mouse (left; *n* = 5 mice from two independent experiments) and human (right; *n* = 5–11 individuals) skin samples; asterisks show results from one-way ANOVA, and number signs show results from cosinor analysis. **c**, Normalized *Ccl21* expression in sorted dermal LECs measured by qPCR. Data are representative of two independent experiments; *n* = 3 mice; one-way ANOVA with Tukey’s post test. **d**, Quantitative imaging of non-permeabilized ears for CCL21; *n* = 5 mice from two independent experiments; Mann–Whitney *U*-test. Scale bar, 50 µm. **e**, Ear CD11c^+^ cells after crawl-in assays and addition of exogenous (exo) CCL21 (left), heparinase (hep) treatment (middle) or anti-CCL21 antibody blockade (right); *n* = 3 mice representative of two independent experiments. **f**, Ear BMDCs after crawl-in assays using *Ccr7*^–/–^ or control cells; *n* = 3 mice representative of two independent experiments; two-way ANOVA with Sidak post test for **e** and **f**. **g**, Velocity of synchronized WT and *Ccr7*^–/–^ BMDCs in collagen migration assays; *n* = 3 mice representative of two independent experiments; Kruskal–Wallis test with Dunn’s post test. **h**, Ear CD11c^+^ cells after crawl-in assays and antibody treatment; *n* = 3 mice representative of two independent experiments; two-way ANOVA with Sidak post test. **i**, Ear CD11c^+^ cells after crawl-in assays using *Cd99*^–/–^ animals (control, dotted lines); *n* = 5 mice from two independent experiments; unpaired Student’s *t-*test. **j**,**k**, Ear CD11c^+^ cells after crawl-in assays and antibody treatments; *n* = 6 mice (from two independent experiments for **j**) and *n* = 3 mice (representative from two independent experiments for **k**); two-way ANOVA with Sidak post test. **l**, Quantitative imaging of CD11c^+^ cell distance to LVs after ear crawl-in assays; Student’s *t*-test; *n* = 314, 451, 450, 453, 374, 408, 394, 396, 374 and 419 cells (left to right) from three mice. Scale bar, 10 µm. Outline represents orthogonal LV view. Dotted lines represent fit cosinor curves. All data are represented as mean ± s.e.m. Source data

**Extended Data Fig. 5:**
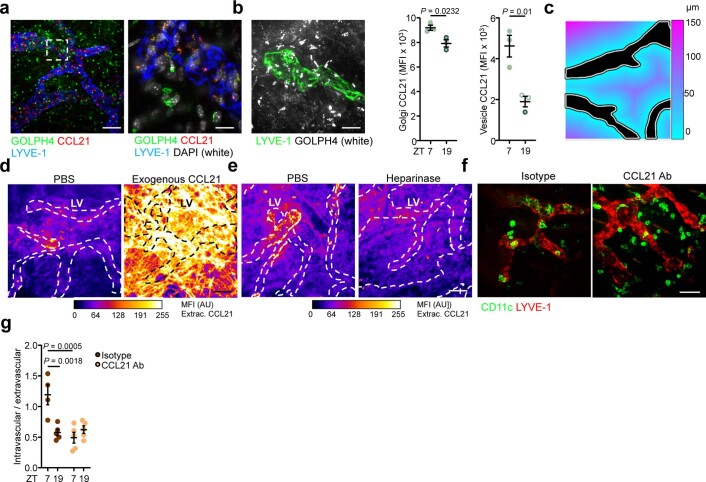
Chemotaxis analyses. **a**, Whole-mount staining of CCL21 in GOLPH4^+^ regions localized in LYVE-1^+^ capillaries with lower (left) and higher (right) magnification. Scale bar, 50 µm and 10 µm, respectively, representative images of 2 independent experiments. **b**, MFI of CCL21 in GOLPH4^high^ (golgi; left) and GOLPH4^low^ (vesicle; right) regions as stained and measured in whole-mounted split ears. Scale bar, 10 µm, unpaired student’s *t*-test, n = 3 mice, data and images are representative of 3 independent experiments. **c**, Generation of distance maps using LYVE-1^+^ capillaries for CCL21 gradient analysis. **d**–**g**, Manipulation of CCL21 gradients, images are representative of 2 independent experiments. **d**, Whole mount staining of non-permeabilized ears for CCL21 after addition of exogenous CCL21. **e**, Visualization of CCL21 MFI in non-permeabilized ears after heparinase or PBS incubation. Dashed lines represent LYVE-1^+^ LV outlines. **f**, 24 h endogenous crawl-in assay after antibody-mediated blockade of CCL21 in comparison with isotype controls and staining for CD11c and LYVE-1 at ZT7. Scale bars, 50 µm. **g**, 3 h BMDC crawl-in assay after pharmacological blockade of CCL21, two-way ANOVA with Tukey’s post test, n = 4,5,5,5 mice from 3 independent experiments. All data are represented as mean ± s.e.m. Source data

Because expression of the chemokine receptor CCR7 was rhythmic in DCs, we investigated the expression of CCL21, the CCR7 ligand, in LECs. In quantitative immunofluorescence imaging analyses of the mouse ear, intracellular expression of CCL21 protein peaked at ZT7 in LECs (Fig. 3a,b). CCL21 also had a rhythmic expression in human skin biopsies, with a peak at 8:00 (Fig. 3b). *Ccl21* mRNA abundance peaked at night (ZT19) in mouse LECs (Fig. 3c), indicating it was phase shifted compared to CCL21 protein and pointing to transcriptional control of its rhythmic expression^24^. Higher CCL21 protein expression was detected at ZT7 (day) than at ZT19 (night) in the Golgi and intracellular vesicles of LECs within mouse skin (Extended Data Fig. 5a,b), indicating diurnal release into the environment. We used quantitative immunofluorescence imaging of non-permeabilized ear whole mounts to resolve the CCL21 gradient in the extracellular interstitial tissue in mice^8^. The use of distance-dependent fluorescence masks (Extended Data Fig. 5c) indicated higher immunoreactivity to CCL21 at ZT7 (day) than at ZT19 (night), particularly in proximity to LVs (Fig. 3d). Elimination of the CCL21 gradient by adding exogenous CCL21, heparinase digestion^8^ or a CCL21 antibody decreased DC migration into LVs at ZT7 compared to control-treated samples and completely abrogated the differences in DC migration between ZT7 (day) and ZT19 (night) (Fig. 3e and Extended Data Fig. 5d–g). *Ccr7*^–/–^ BMDCs exhibited strongly reduced migration into LVs and a lack of rhythmicity compared to BMDCs generated from control mice (Fig. 3f). Synchronized *Ccr7*^–/–^ BMDCs at CT24 and CT36 exhibited no differences in migration velocity in collagen gel chemotaxis assays compared to synchronized wild-type (WT) control BMDCs (Fig. 3g), indicating that the circadian expression of CCR7 controlled the rhythmicity of DC migration.

**Extended Data Fig. 6:**
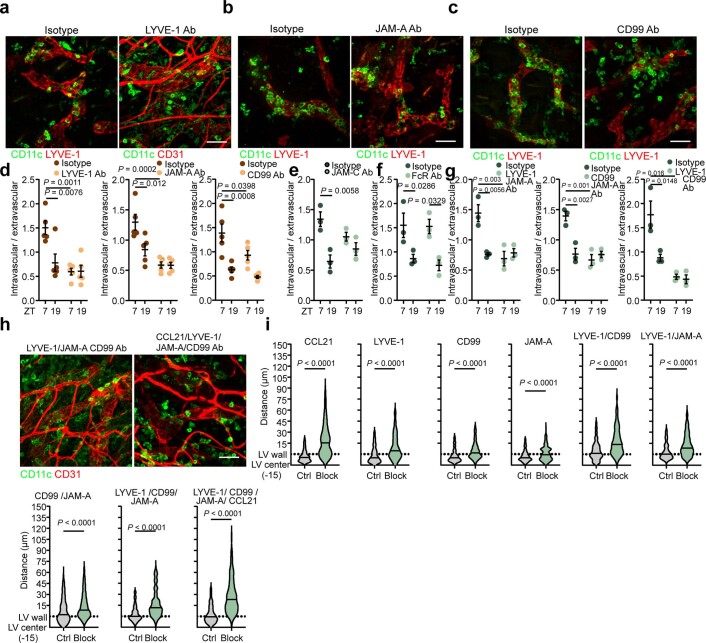
Adhesion and transmigration analysis. **a**–**c**, 24 h endogenous crawl-in assays after antibody-mediated blockade of LYVE-1, **a**, JAM-A, **b**, or CD99, **c**, and respective isotype controls, stained for CD11c, CD31 or LYVE-1, representative of 2 independent experiments. Scale bars, 50 µm. **d**, 3 h BMDC crawl-in assays after antibody-mediated blockade of LYVE-1 (left), JAM-A (centre) and CD99 (right). n = 5 mice from 2 independent experiments. **e**, **f**, Neutralization of JAM-C (**e**) and Fc receptor (FcR, **f**) prior to a 24 h endogenous crawl-in assay with n = 3 mice. **g**, Combinations of anti-LYVE-1, anti-JAM-A and anti-CD99 for double blocks prior to a 24 h endogenous crawl-in assay, n = 3 mice, data are representative of 2 independent experiments, two-way ANOVA with Šidák post test. **h**, 24 h endogenous crawl-in assay with a combined antibody blockade of LYVE-1, CD99, JAM-A without CCL21 (left) or with CCL21 (right), stained for CD11c and CD31 representative of 2 independent experiments. Scale bar, 50 µm. **i**, Distance of CD11c^+^ cells to LV centre after 24 h crawl-in assays and antibody-mediated migration ablation or isotype controls at ZT7. Shown are individual blocks with respective controls, student’s *t*-test, n = 364–504 cells from 3 mice. All data are represented as mean ± s.e.m. Source data

Antibody-mediated blockade of LYVE-1, CD99 and JAM-A in ear explant assays resulted in a marked decrease in CD11c^+^ DC immigration into skin lymphatics at ZT7 (day) compared to isotype-treated controls, whereas no effects were observed in DC immigration at ZT19 (night) (Fig. 3h and Extended Data Fig. 6a–d). JAM-C blockade ablated diurnal variation but did not reduce DC immigration into afferent lymphatics at ZT7 or ZT19 (Extended Data Fig. 6e), whereas Fc receptor blockade had no effect on DC trafficking into LVs (Extended Data Fig. 6f). The relevance of CD99 in lymphatic migration was confirmed with *Cd99*^–/–^ mice (Fig. 3i). Because individual blocking approaches abolished the diurnal differences in DC migration and blocked migration into the afferent lymphatics only at ZT7 (day) but not ZT19 (night), we tested whether these signals act redundantly. Combined blockade of LYVE-1, CD99 and JAM-A reduced the migration of DCs into LVs at both ZT7 and ZT19, while additional CCL21 blockade completely inhibited DC infiltration into afferent lymphatics at both time points (Fig. 3j,k and Extended Data Fig. 6g,h). We used distance and distribution analyses to quantify at which step in the rhythmic migration process each molecule was required. Blockade of LYVE-1, CD99 or JAM-A arrested DCs at the level of adhesion to the LEC. By contrast, blocking CCL21 retained the DCs in the interstitial area (Fig. 3l and Extended Data Fig. 6i), indicating that the migration of DCs is tightly controlled by successive interactions of promigratory factors.

**Fig. 4:**
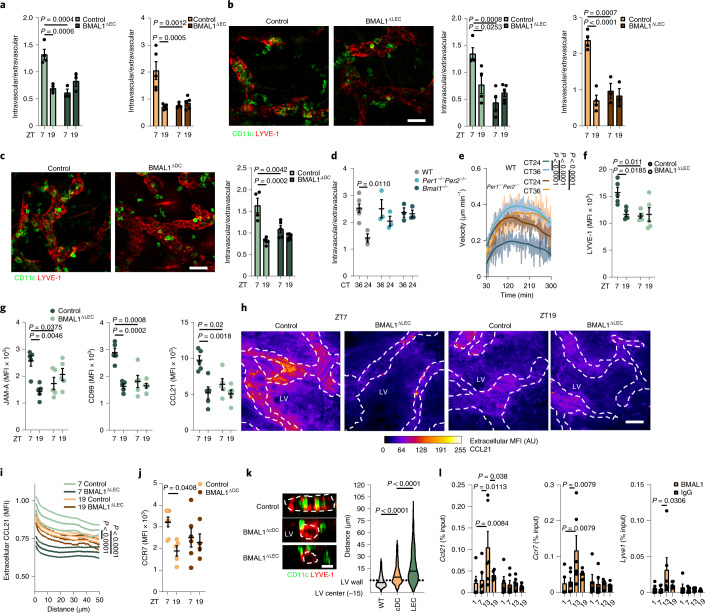
Lineage-specific BMAL1 deficiency abrogates rhythms in DC lymphatic trafficking. **a**–**c**, Ear CD11c^+^ cells (green) or BMDCs (orange) after crawl-in assays with lineage-specific *Bmal1*^–/–^ animals; *n* = 4, 4, 3 and 3 mice (left) and 5, 5, 4 and 5 mice (right) (**a**); *n* = 4, 4, 4 and 5 mice (left) and 4, 4, 3 and 3 (right) (**b**); *n* = 4, 4, 5 and 5 mice (**c**); data are representative of two independent experiments; two-way ANOVA with Sidak post test. **b**,**c**, Representative ear whole-mount images from control and BMAL1^ΔLEC^ (**b**) or BMAL1^ΔcDC^ (**c**) animals. Scale bar, 50 µm. **d**, Ear BMDCs after crawl-in assays with synchronized control, *Per1*^–/–^*Per2*^–/–^ or *Bmal1*^–/–^ BMDCs; *n* = 6 mice for WT cells and *n* = 3 for knockout (KO) cells, representative of two independent experiments; two-way ANOVA with Sidak post test. **e**, Velocity of synchronized WT and *Per1*^–/–^*Per2*^–/–^ BMDCs in collagen gel migration assays; *n* = 3 mice. Data are representative of two independent experiments; Kruskal–Wallis test with Dunn’s post test. **f**,**g**, Quantitative immunofluorescence screen of LYVE-1 (**f**), JAM-A, CD99 and CCL21 (**g**) in LYVE-1^+^ ear LVs; *n* = 5 mice from two independent experiments; two-way ANOVA with Sidak post test. **h**,**i**, Ear whole-mount imaging (**h**) and quantification (**i**) of CCL21 of WT and BMAL1^ΔLEC^ ears; *n* = 5 mice from two independent experiments; Kruskal–Wallis test with Dunn’s post test. Dashed lines represent LYVE-1^+^ capillaries. Scale bar, 50 µm. **j**, Flow cytometric analysis of CCR7 on CD11c^+^MHCII^+^CD103^–^EpCAM^–^ DCs in control and BMAL1^ΔcDC^ animals; *n* = 7, 5, 6 and 4 mice from two independent experiments; two-way ANOVA with Sidak post test. **k**, Representative images and quantification of the distance of CD11c^+^ cells to LVs after ear crawl-in assays; *n* = 340, 431 and 575 cells from four mice from two independent experiments; unpaired Student’s *t*-test. Scale bar, 10 µm. Outline represents orthogonal LV view. **l**, Chromatin immunoprecipitation (ChIP) of BMAL1 binding to promoter regions of *Ccl21*, *Ccr7* and *Lyve1* compared to IgG controls; *n* = 6 mice from two independent experiments; two-way ANOVA with Sidak post test. All data are represented as mean ± s.e.m. Source data

**Extended Data Fig. 7:**
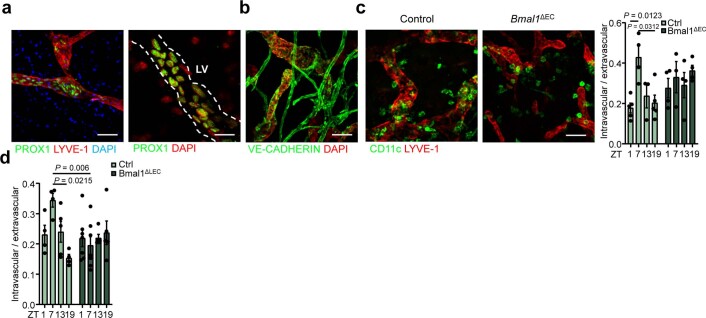
Lineage-specific BMAL1-deficiency migration analysis. **a**, **b**, Representative whole mount staining for PROX-1 and VE-CADHERIN in mouse EAR skin. Dotted lines represent a LYVE-1^+^ capillary. Scale bars, 50 µm and 10 µm for higher magnification. **c**, **d**, 6 h endogenous crawl-in assays in EC-specific (*Bmal1*^ΔEC^; **c**) and LEC-specific (*Bmal1*^ΔLEC^; **d**) *Bmal1*^-/-^ animals and controls. Values presented as intravascular versus extravascular ratio of CD11c^+^ cells. Representative images show a 24 h endogenous crawl-in assay, stained for CD11c and LYVE-1, scale bar, 50 µm, two-way ANOVA with Šidák post test, n = (c, WT) 5,4,3,5; (c, KO) 4,3,4,4); (d, WT) 4,4,5,4; (d, KO) 7,7,5,5) mice from 2 independent experiments. All data are represented as mean ± s.e.m. Source data

Finally, we assessed the role of circadian clock genes in endothelial cells and DCs in controlling DC migration from the skin. DCs from *Cdh5cre*^ERT2^*Bmal1*^flox^ mice, in which the circadian transcription factor BMAL1 is deleted in blood endothelial cells and LECs (BMAL1^ΔEC^), and *Prox1cre*^ERT2^*Bmal1*^flox^ mice, in which BMAL1 is deleted in LECs (BMAL1^ΔLEC^) (Extended Data Fig. 7a,b), showed no rhythmic migration into skin LVs in ears collected at ZT7 and ZT19 compared to *Bmal1*^flox^ control mice (Fig. 4a,b and Extended Data Fig. 7c,d). In addition, *Clec9acreBmal1*^flox^ mice, which lack BMAL1 in cDCs (BMAL1^ΔcDC^), showed significantly reduced and non-rhythmic DC migration into LVs in ear skin explants compared to *Bmal1*^flox^ control mice (Fig. 4c). Furthermore, synchronized BMDCs generated from *Per1*^–/–^*Per2*^–/–^ or *Bmal1*^–/–^ mice had no rhythmic migration behavior when cells were incubated with ears, in contrast to synchronized WT BMDCs (Fig. 4d,e). These observations indicate that the circadian clock proteins in both LECs and DCs are required for DC rhythmic migration into LVs.

**Extended Data Fig. 8:**
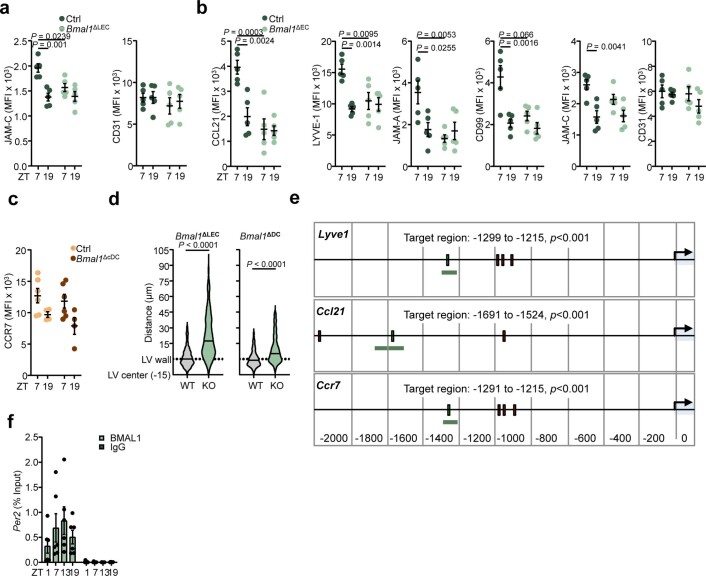
Lineage-specific BMAL1-deficiency protein analysis and ChIP. **a**, **b**, Quantitative immuno-fluorescence microscopy profiling of proteins on LV using 10 µm organ slices in LEC-specific (*Bmal1*^ΔLEC^; **a**) or EC-specific (*Bmal1*^ΔEC^ ; **b**) *Bmal1*^-/-^ and control animals, two-way ANOVA with Šidák post test, n= 5 mice from 2 independent experiments. **c**, Mean fluorescence intensity (MFI) of CCR7 on the surface of Langerhans cells after a 24 h crawl-out assay using cDC-specific (*Bmal1*^ΔcDC^) *Bmal1*^*-/-*^ or control animals n = 4–6 mice from 2 independent experiments. **d**, Distance of CD11c^+^ DCs to LV centre after a 24 h crawl-in assay with tissue-specific *Bmal1*^-/-^ animals. * = unpaired student’s *t*-test, n = (*Bmal1*^ΔLEC^) 371 and 305 cells from 4 mice; (*Bmal1*^ΔcDC^) 394 and 315 cells from 5 mice and 2 independent experiments. **e**, Schematic overview of promoter regions of target genes *Lyve1*, *Ccl21a* and *Ccr7* in LN samples for chromatin immunoprecipitation (ChIP) analysis. The *p*-value marks the probability of the green-indicated BMAL1 binding sides, promoter regions are taken from the eukaryotic promoter database, EPFL, Switzerland. **f**, ChIP qPCR analysis of BMAL1 binding to promoter regions of *Per2* as control at different time points throughout the day compared to IgG controls, n = 6 mice from 2 independent experiments. All data are represented as mean ± s.e.m. Source data

Next, we profiled the expression of promigratory factors in skin LECs by quantitative immunofluorescence. BMAL1^ΔEC^ and BMAL1^ΔLEC^ mice exhibited non-rhythmic LEC expression in CCL21, LYVE-1, CD99, JAM-A and JAM-C, while expression of the non-rhythmically expressed molecule CD31 was not affected (Fig. 4f,g and Extended Data Fig. 8a,b). In addition, the time-of-day differences in the gradient of CCL21 expression were lost in these mice (Fig. 4h,i). cDCs from BMAL1^ΔcDC^ mice did not exhibit rhythmic expression of CCR7, while CCR7 expression in BMAL1^ΔcDC^ LCs was normal compared to LCs collected from *Bmal1*^flox^ control mice (Fig. 4j and Extended Data Fig. 8c). Distance and distribution imaging analyses indicated that DCs arrested more distant to LECs in the interstitial area of BMAL1^ΔLEC^ mice than of BMAL1^ΔcDC^ mice (Fig. 4k and Extended Data Fig. 8d), indicating that lack of endothelial cell BMAL1 expression resulted in a more marked loss of DC migration into LVs than loss in DC BMAL1. These data and the presence of canonical E-box-binding sites for BMAL1 in the promoter regions of *Ccl21*, *Ccr7* and *Lyve1* genes (Extended Data Fig. 8e) indicated that these genes are directly regulated by the circadian clock. ChIP assays detected the rhythmic binding of BMAL1 to these promoters, with a peak at ZT13 (Fig. 4l and Extended Data Fig. 8f), indicating that BMAL1 directly controls the signals governing DC migration into the afferent LVs.

In summary, we present here evidence that the migration of DCs into skin lymphatics is a rhythmic process. We found that CCL21, CCR7 and LYVE-1 are under the direct control of the circadian clock gene *BMAL1*, implicating the clock as an essential and broad regulator of DC migration. As this process is fundamental for the generation of adaptive immunity, it should prove useful to exploit in vaccination and immunotherapeutic regimens, considering that rhythmicity in innate and adaptive immune responses is maintained in inflammatory reactions^13,14,21,25–27^. We also observed analogous but phase-shifted rhythmicity in the expression of human promigratory factors, suggesting that these functional diurnal differences observed in mice should also apply to humans.

## Methods

### Mouse models

Male WT C57BL/6N mice aged 6–8 weeks were purchased from either Charles River Laboratories or Janvier labs. *Cdh5-creERT2* (B6) mice were obtained as a gift from R. Adams (Max Planck Institute for Molecular Biomedicine) and crossbred with *Bmal1*^flox/flox^ (B6) mice obtained from Jackson Laboratories to target blood endothelial cells and LECs. *Prox1-creERT2* (B6) mice (Jackson Laboratories) were crossbred with *Cdh5-creERT2-Bmal1*^flox/flox^ mice to obtain *Prox1-creERT2-Bmal1*^flox/flox^ mice to specifically target LECs. *Cdh5-creERT2* and *Prox1-creERT2* mice were given intraperitoneal tamoxifen injections for five consecutive days at the age of 6 weeks to induce Cre recombinase expression and excise *Bmal1* at the respective flox regions. *EYFP*; *Clec9acre* mice were crossbred with *Bmal1*^flox/flox^ mice to target cDCs^28^. *Cd99*^–/–^ mice and bone marrow (BM) cells from *Ccr7*^–/–^ mice, *Bmal1*^–/–^ mice and *Per2*::*Luc* mice aged 6–8 weeks were used. Primary BM cells from *Per1*^–/–^*Per2*^–/–^ mice were provided by J. Ripperger and U. Albrecht (University of Fribourg).

All animals were housed under a 12-h light/12-h dark schedule with ad libitum access to water and food in the Core Facility Animal Models at the Biomedical Centre (Ludwig-Maximilians-Universität) or the University of Geneva. To facilitate simultaneous experiments at different time points of the day, light cabinets (Techniplast) were used to shift animals to different light–dark cycles. For experiments requiring altered lighting patterns, mice were either housed in complete darkness or given 2 weeks to adapt to inverted lighting patterns, respectively. All animal procedures and experiments were in accordance with the ministry of animal welfare of the region of Oberbayern and with the German law of animal welfare or were approved and performed in accordance with the guidelines of the animal research committee of Geneva.

### Human skin biopsy sampling

Skin biopsies were taken from male and female adults (mean age, 74 years) presenting at the dermatosurgery unit of the Geneva University Hospitals for the removal of skin tumors under local anesthesia. Written informed consent was obtained from each individual. Sampling was conducted according to the Declaration of Helsinki and approved by the Commission Cantonale d’Ethique de la Recherche of the University Hospitals of Geneva. Samples were taken from excessive, tumor-free surrounding skin known as ‘Burow’s triangles’ during the reconstruction of dermatosurgical excisions. No extra incisions were made to obtain the sample, and the size of the original tumor excision was not altered by this study. The sample was placed into normal saline solution immediately after it had been excised, and the time of day was noted. Samples were then embedded in optimal cutting temperature compound (OCT) and shock frozen until sectioning.

### BMDC cell culture, synchronization and labeling

BMDCs used for exogenous crawl-in assays were cultured using R10 medium (RPMI, 10% fetal calf serum (Gibco), 2 mM l-glutamine, 1% penicillin/streptomycin and 50 µM β-mercaptoethanol) supplemented with 20 ng ml^–1^ granulocyte–macrophage colony-stimulating factor (GM-CSF) hybridoma supernatant. GM-CSF-producing hybridoma cells were used as described^29^. GM-CSF concentration in the supernatant was measured using an mGM-CSF Quantikine ELISA kit (R&D Systems).

Cells were cultured for 10 d. At day 10, all non-adherent cells were collected and immersed in R10 medium supplemented with 10 ng ml^–1^ GM-CSF and stimulated with 100 ng ml^–1^ LPS (Sigma-Aldrich) for 24 h. Viability and purity of BMDCs were checked by analysis of MHCII and CD11c expression on DAPI-negative BMDCs.

To synchronize BMDCs, on day 8, 7 × 10^6^ BMDCs per sample culture were replated. The next day, BMDCs were placed in 50% horse serum (Merck, H1138) for 2 h (shock)^20,22^ with two subsequent washes and on day 10 received the LPS challenge.

After maturation and stimulation, the required number of cells was incubated with 5 µM CellTrace Violet or CellTrace Yellow (1:1000; Thermo Fisher), thoroughly washed and adjusted to a final concentration of 5–7.5 × 10^5^ cells per ml to be used for migration assays.

### Luciferase-expressing BMDCs

For BMDC synchronization experiments, BM was collected from *Per2*::*Luc* mice and grown as described above. On day 8, all non-adherent cells were collected and resuspended at 2 × 10^5^ cells per ml in complete medium. Cells were replated into 35-mm dishes (2 ml per dish) and left overnight to recover. To synchronize, cells were incubated in complete medium with 50% horse serum for 2 h. Following synchronization, cells were washed gently with complete medium to remove excess serum and left in 2 ml of fresh medium containing 100 µM luciferin (BioLume) and LPS. Dishes were sealed with parafilm and transferred to a LumiCycle (Actimetrics) for bioluminescence recording. Photon counts per minute were detrended using a 24-h moving average to calculate relative bioluminescence.

### Exogenous BMDC crawl-in migration assay

For exogenous BMDC crawl-in migration assays, mice were killed according to animal protocols. Protocols were adapted^17^, ears were carefully collected and their dorsal and ventral sides were separated and quickly rinsed in medium. Ears were placed in custom-built imaging chambers and immobilized. Labeled BMDCs (25,000–37,500) were added onto ears for 10–15 min. After washing off any unbound BMDCs, ears were completely covered with R10 medium and incubated for a maximum of 3 h. Ear whole mounts were stained with different combinations of LYVE-1, CD31 or podoplanin and were subsequently fixed in 4% paraformaldehyde. Images of ears were obtained as whole mounts using a Zeiss Axio Examiner.Z1 confocal spinning disk microscope equipped with 405-, 488-, 561- and 640-nm laser sources.

For end-point analysis, at least eight images per ear whole mount were taken from LVs (precollecting and capillaries). The number of cells outside or inside the vessels was counted using three-dimensional (3D) visualization tools in Fiji and Slidebook 6.0 as well as orthogonal views and optical slicing. This number was then normalized to the calculated volume of the vessels.

For short crawl-ins, distance-dependent zone segmentation of the interstitial area and accumulation analyses were used. After BMDC addition and wash-off, ears were incubated in medium for 0–60 min, stained and imaged as described above. In each *z* projection, CD31^+^ LVs were manually outlined, a binary mask was generated and LV distance-dependent maps were applied to create specific segments in the interstitial area using a custom-made script in Matlab. BMDCs were manually counted within these segments, and the relative distribution was calculated.

### Endogenous skin DC mobilization and crawl-in assay

For endogenous leukocyte crawl-in migration assays, mice were killed according to animal protocols, and collected ears were split. After an initial wash, ear halves were stored on medium with the dermis facing down for 6 h or 24 h. Afterwards, ear sheets were fixed in 4% paraformaldehyde and stained for CD11c, LYVE-1 and/or CD31. Cells were counted and normalized, and the in-versus-out ratio was generated as described above.

### Live imaging of BMDC crawl-in assays

For live imaging of exogenous crawl-in assays, ear whole mounts were prepared as described above; however, they were subjected to an initial staining before being used for the trafficking assay. Ears were stained for laminin with a primary antibody and then probed with a secondary FITC-conjugated antibody in medium at room temperature before receiving 50,000–75,000 BMDCs for 10 min. Unbound BMDCs were washed off, and the ear half was immersed in phenol red-free R10 medium supplemented with 10 mM HEPES (Sigma). Imaging was performed in a 5% CO_2_ chamber at 37 °C with one 30–40-µm-deep 3D image acquired every 100 s for 45–60 min. Migration paths, distance plots and velocity, directionality, Euclidian and accumulated distances were calculated using the TrackMate and manual tracking plugins in Fiji together with the Ibidi chemotaxis and migration tool.

### Functional blocking of proteins and abrogation of chemokine gradients

To investigate the functional relevance of proteins involved in rhythmic dermal DC trafficking, antibodies or enzymes were used. Ears of exogenous crawl-in assays were incubated with medium containing diluted neutralization antibodies before the migration assay for 1 h at room temperature or directly added to the endogenous crawl-in medium for 24 h (see antibody list in the Supplemental Information).

For abrogating the extracellular CCL21 gradient, ears were incubated with either heparinase II and IV (100 mIU; Sigma) at 37 °C for 1 h or placed in PBS containing 0.1% bovine serum albumin and 0.6 µg ml^–1^ CCL21 (Peprotech) at 4 °C for 90 min before the migration. Unbound CCL21 or remaining heparinase was washed off afterwards.

### Lymphatic distance analysis

To measure the distance between DCs and the LV, a combination of orthogonal views, maximum *z* projections and 3D viewers was used. At least 250 CD11c^+^ cells per biological replicate were taken for analysis. To calculate the average diameter of LVs, 40 vessels were used as a proxy. The distance between LV and DC center was then manually measured using Fiji.

### Crawl-out assay

Collected and split ears were washed for 2 h and stored in R10 medium for 24 h supplemented with 1 µg ml^–1^ CCL21. The next day, ears were collected and gently digested for 20 min at 37 °C with collagenase IV (1 mg ml^–1^; C5138, Sigma), DNase (0.2 mg ml^–1^; Roche) and dispase II (0.2 mg ml^–1^; Sigma). After digestion, cells were filtered through a 70-µm cell strainer, washed and resuspended in PBS supplemented with 2% fetal calf serum and 2 mM EDTA (Sigma). Simultaneously, the medium containing the emigrated DCs was collected, and both the ear DC and medium DC populations were first Fc receptor blocked with anti-mouse CD16/CD32 for 5 min at room temperature and subsequently stained with fluorescence-conjugated antibodies for 30 min at 4 °C. For CCR7, the stain was performed separately at 37 °C before the other staining step. DAPI and full-bright counting beads (Thermo Fisher) were added to cells, which were then analyzed by flow cytometry using a Gallios flow cytometer (Beckman Coulter) equipped with 405-, 488- and 633-nm lasers or a BD Fortessa flow cytometer (405-, 488-, 561- and 633-nm lasers; BD Biosciences).

### In vitro DC migration assay

Synchronized BMDCs (500,000) were washed and used for in vitro chemotaxis migration assays as described^30,31^. For preparation of the collagen gel mixture, 30 µl of 10× MEM and 15 µl of 7.5% sodium bicarbonate were mixed until the solution turned pink. Next, 225 µl of Nutragen (purified bovine collagen I solution 6 mg ml^–1^) was added and mixed homogenously. Two hundred microliters of this collagen gel mixture and 100 µl of the cell suspension were mixed carefully to avoid the formation of air bubbles. Two hundred and eighty microliters of the mixture was pipetted into custom-made chambers. After polymerization for 75 min at 37 °C and 5% CO_2_, gels were overlaid with 80 µl of CCL21 (1 µg ml^–1^). Afterwards, the chambers were sealed with paraffin.

For imaging of cell migration, one picture per condition was recorded every 60 s for 5 h with an inverted wide-field Leica DMi8 microscope using a ×4 objective, a Leica Lumencor SpectraX multi-LED light source, an incubation chamber set to 37 °C and a heated stage.

For cell migration analysis, the first 30 frames of the recordings were excluded due to initial image instability caused by technical reasons. Frames 31 to 300 were analyzed in ImageJ using a custom-made script^32^.

### Topical FITC ear stimulation

FITC (8 mg ml^–1^; Sigma) in 1:1 dibutyl phthalate (Sigma) and acetone (Sigma) was thoroughly vortexed before use. Mice were anesthetized by isoflurane inhalation, and 25 µl of this solution was applied to the ear skin and left for 24 h, after which ears were collected for imaging analyses.

### Immunofluorescence staining

To measure the expression level of adhesion molecules in LECs, mouse organs (skin, inguinal LN, lung and small intestine) were collected at four time points, placed in OCT (TissueTec), shock frozen on dry ice and stored at −80 °C. The next day, organs were thawed to −20 °C and sectioned at a thickness of 10 µm on a cryostat (Leica)^26^. Tissue sections were stored at −80 °C or were directly thawed to room temperature and encircled with a hydrophobic alcohol-resistant pen. Sections were fixed with cold methanol (100%; Sigma) for 10 min, washed, blocked and permeabilized in PBS containing Triton X-100 (0.5%; Sigma) and normal goat serum (20%; Sigma). Sections were always stained for LYVE-1 in combination with the proteins of interest (see antibody list in the Supplementary Information) overnight at 4 °C. The next day, samples were washed with PBS and imaged.

To stain CCL21 on tissue slides, the blocking step was expanded using avidin (20%). Slides were thoroughly washed and incubated in PBS containing 20% biotin (Vector Labs), and sections were stained for LYVE-1 and CCL21-biotin overnight at 4 °C. The next day, samples were washed with PBS and incubated in labeled streptavidin (2.5 µg per slice) at room temperature for 15 min.

For quantification, five images of different regions of one section from one mouse were acquired, averaged and counted as one biological replicate. The average MFI of five isotype stainings was deducted from the MFI of the protein staining of interest. The MFI was then normalized to the second highest value of all ZTs. Fold change values of MFI between ZTs were generated and shown as a heat map.

### Whole-mount immunofluorescence staining

To better understand the 3D architecture of proteins expressed on or in LECs, ears were whole mounted and 3D imaged. Fixation of ears was performed before or after staining with antibodies. In the case of staining for CD31 or PROX-1, fixation was done after staining. If staining for LYVE-1, podoplanin, laminin, CCL21, GOLPH4, VE-cadherin or CD11c, ears were fixed before staining.

For staining of GOLPH4 and CCL21, ears were washed the next day and stained using secondary antibodies for 2 h at room temperature. Imaging of whole mounts was performed as described above.

### CCL21 gradient analysis

To visualize and analyze the extracellular CCL21 gradient, collected and fixed ears were carefully washed and blocked in R10 medium containing avidin (20%) for 1 h as previously described^8^. After leaving the ears in PBS and biotin (20%) for 30 min, they were stained for LYVE-1 and CCL21 (Biotin) overnight at 4 °C. The next day, ears were incubated in PE-labeled streptavidin for 3 h at 4 °C in the dark and imaged.

Intensities of interstitial CCL21 chemokine signals were quantified on maximum intensity projections ranging between 30- and 40-µm *z* stacks with a *z* step size of 1 µm. Using custom-written Matlab scripts, LV capillaries were manually outlined by an experimentor blinded to the respective experimental condition and converted into binary masks. Next, a Euclidean distance transform was computed on each binary mask, resulting in the shortest Euclidean distance of each non-masked pixel to its nearest mask border/LV. Euclidean distances were rounded and translated into metric units via the microscope’s embedded pixel resolution. The distance masks were then applied to their respective CCL21 stainings, and fluorescent intensities were extracted and averaged across distances, resulting in a distance-dependent fluorescent intensity metric per image. Average intensity images of CCL21 staining were integrated into distance-dependent fluorescence intensities. Five average distance-dependent fluorescence intensities from different locations within one mouse ear were averaged as one biological replicate. All samples from ZT7 and ZT19 were normalized to the highest average fluorescence intensity of ZT7. Isotype stainings were taken as negative controls.

### CCL21 localization analysis

To localize CCL21 intracellularly, ears were fixed, permeabilized and blocked as mentioned above. After staining for CCL21, LYVE-1 and GOLPH4, using primary and appropriate secondary antibodies, at least five different regions per ear were imaged. Thirty- to forty-micron *z* stacks with a step size of 1 µm were *z* projected and analyzed using Fiji. CCL21 signal intensity was quantified in LYVE-1^+^ cells in either GOLPH4^high^ or GOLPH4^low^ regions. The MFI of isotype controls was then deducted from the CCL21 staining.

### Sorting of LECs

To sort dermal LECs for RNA sequencing, four ears from two mice were pooled per biological replicate and time point. Dermal cells were isolated as stated above and prepared for sorting on a FACSAria IIIu (BD) equipped with four lasers (405, 488, 561 and 633 nm) in the Core Facility Flow Cytometry at Biomedical Centre Munich. Live podoplanin^+^CD31^+^ LECs were sorted directly into either 350 µl of TriZol-LS (Thermo Fisher in the case of RNA sequencing) or 350 µl of RLT buffer with β-mercaptoethanol (1:100) (Qiagen and Sigma, respectively, in the case of qPCR) at 4 °C using a 100-µm nozzle and a purity of >90% as determined by purity checks after every sort. Cell numbers sorted ranged between 3,000 and 5,000 cells per two pooled ear pairs. Directly after sorting, samples were shock frozen on dry ice and stored for further analysis.

#### RNA sequencing

RNA of sorted dermal LECs collected in TriZol-LS was purified using a Direct-zol RNA mini prep kit (ZymoResearch) following the manufacturer’s instructions. Isolated RNA was quantified using a Nanodrop (Thermo Fisher) and analyzed on a Bioanalyzer (Agilent) using an RNA 6000 Nano or Pico kit (Agilent). Eluted total RNA (75 ng) was digested with DNase (Thermo Fisher) to remove DNA contamination. An additional purification step with RNAClean XP beads (Agencourt) was performed. The purified, bead-bound RNA was directly used as input in the SMART-Seq v4 Ultra Low Input RNA kit (TaKaRA Bio). Full-length cDNA was generated following the manufacturer’s instructions. Full-length cDNA was quantified using a Qubit dsDNA HS Assay kit (Invitrogen) with a Qubit fluorometer. Finally, sequencing libraries were generated using 500 pg of full-length cDNA following the NexteraXT protocol (Illumina). Libraries were quantified on a Bioanalyzer (Agilent) using a DNA 1000 kit (Agilent) and sequenced on a HiSeq1500 system (Illumina) with a read length of 100 nucleotides in single-end mode.

For data processing, obtained transcriptome profiles were processed on a Galaxy web interface hosted by LAFUGA (Gene Center). After demultiplexing and trimming, data were mapped against the mouse genome (mm10) using RNA-STAR mapper (Galaxy version 2.5.2b-0). Abundant reads were counted using HTSeq-count (Galaxy version 1.0.0). Afterwards, gene expression analysis to detect differentially expressed genes was performed using DESeq2 (Galaxy version 2.11.40.6), setting the false discovery rate to <0.05. The sequencing data have been deposited in the NCBI Gene Expression Omnibus (GEO) and are accessible through GEO series accession number GSE184758.

#### GO cluster enrichment analysis

GO cluster enrichments were generated using metascape.org (ref. ^33^). Enriched genes from the DESeq2 analysis with *P* ≤ 0.05 were organized in gene lists and screened for GO biological process enrichment. A *P* value of ≤0.01, a minimum count of 3 and an enrichment factor of ≥1.5 were used as cutoff parameters. Genetic terms were collected and grouped into clusters based on their membership similarities.

To compare the biological adhesion (GO:0022610) gene counts, sequencing results were filtered for GO:0022610 cluster genes, and significantly up- and downregulated genes were compiled in a heat map.

#### qPCR

RLT-sorted LECs or lysed synchronized BMDCs were thawed and thoroughly mixed. RNA was isolated using the RNeasy micro kit (Qiagen) according to the manufacturer’s instructions. RNA samples were analyzed using a NanoDrop2000 (Thermo Scientific) to determine RNA concentration and quality. Obtained RNA was directly reverse transcribed into cDNA with the High Capacity cDNA Reverse Transcription kit (Applied Biosystems). cDNA samples were stored at −20 °C before use. qPCR was performed with a StepOnePlus Real-Time PCR System (Applied Biosystems) in 96-well plates with SYBR green-compatible primers. Each qPCR sample was analyzed in duplicate. The total reaction volume was 10 µl and contained 5 µl of SYBR green, 1 µl of primer mix (5 µM), 3 µl of water and 1 µl of cDNA. Gene expression levels were normalized to the housekeeping gene *Rpl32*.

#### ChIP analyses

After animals were killed, draining LNs were snap frozen on dry ice and stored at −80 °C before processing. Tissue was dounce homogenized in 1 ml of homogenization buffer (10 mM HEPES-KOH, 10 mM KCl, 5 mM MgCl_2_, 0.5 mM DTT and 1× Complete Protease Inhibitor (Roche)). The homogenate was fixed in PBS containing 1% final formaldehyde (Thermo Fisher Scientific). Nuclei were isolated, and chromatin suspensions were obtained through sonication (Diagenode Bioruptor) to obtain fragments of 0.2–0.8 kilobases in size. Immunoprecipitation was performed with anti-BMAL1 (D2L7G, Cell Signaling Technology) or control IgG (Abcam). DNA was isolated with MinElute PCR Purification kits (Qiagen). DNA concentration was determined by using a Qubit dsDNA HS kit (Thermo Fisher Scientific). Real-time ChIP–qPCR was performed by use of SYBR Green Master Mix (Roche) in a LightCycler 480 II (Roche). Occupancy of BMAL1 at the *Ccr7*, *Ccl21*, *Lyve1* and *Per2* promoters was quantified by qPCR targeting regions identified as containing E-boxes using the SCOPE motif finder and EPFL eukaryotic database. Relative enrichment was determined as percentage of the input.

### Statistics

All figures represent merged data from independent biological replicates, and exceptions are explained in the figure legends. Adequate control experiments were individually performed for each biological replicate. Validation of replicates was performed independently, and they were pooled only when all replicates demonstrated invariable results. Complete statistical analyses, including the cosinor test, were done using GraphPad Prism, taking into consideration normal and non-normal data distributions; ^*^ or ^#^, *P* < 0.05; ^**^ or ^##^, *P* < 0.01; ^***^ or ^###^, *P* < 0.001; ^****^ or ^####^, *P* < 0.0001.

### Reporting Summary

Further information on research design is available in the Nature Research Reporting Summary linked to this article.

## Online content

Any methods, additional references, Nature Research reporting summaries, source data, extended data, supplementary information, acknowledgements, peer review information; details of author contributions and competing interests; and statements of data and code availability are available at 10.1038/s41590-021-01040-x.

## Supplementary information

Supplementary Information Supplementary information on antibodies, materials and software.

Reporting Summary

Supplementary Video 1 Video 1. Exogenous crawl-in ZT7. Live imaging of a ZT7 ear for the time indicated. BMDCs are stained in green, and basal membrane staining (laminin) is shown in red; scale bar, 100 µm. Time is in h:min:s. Abbreviations: ZT, zeitgeber time; BMDC, bone marrow-derived dendritic cells.

Supplementary Video 2 Video 2. Exogenous crawl-in ZT19. Live imaging of a ZT19 ear for the time indicated. BMDCs are stained in green, and basal membrane staining (laminin) is shown in red; scale bar, 100 µm. Time is in h:min:s. Abbreviations: ZT, zeitgeber time; BMDC, bone marrow-derived dendritic cells.

Supplementary Video 3 Video 3. Exogenous crawl-in using synchronized BMDCs. Live imaging of a ZT7 ear for the time indicated. BMDCs are differentially stained and added on top of the same ear. CT36 BMDCs are stained in red, CT24 BMDCs are stained in green and basal membrane staining (laminin) is shown in blue; scale bar, 100 µm. Time is in h:min:s. Abbreviations: ZT, zeitgeber time; CT, circadian time; BMDC, bone marrow-derived dendritic cells.

Supplementary Tables Supplementary Tables 1–3.

## Source data

Source Data Fig. 1 Statistical and imaging source data.

Source Data Fig. 2 Statistical and imaging source data.

Source Data Fig. 3 Statistical and imaging source data.

Source Data Fig. 4 Statistical and imaging source data.

Source Data Extended Data Fig. 1 Statistical and imaging source data.

Source Data Extended Data Fig. 2 Statistical and imaging source data.

Source Data Extended Data Fig. 3 Statistical and imaging source data.

Source Data Extended Data Fig. 4 Statistical and imaging source data.

Source Data Extended Data Fig. 5 Statistical and imaging source data.

Source Data Extended Data Fig. 6 Statistical and imaging source data.

Source Data Extended Data Fig. 7 Statistical and imaging source data.

Source Data Extended Data Fig. 8 Statistical and imaging source data.

## Data Availability

All data that support the conclusions of this paper are available at 10.26037/yareta:alphbk7uinejtgflybg7utlasq. Source data are provided with this paper. The sequencing data have been deposited in the NCBI GEO and are accessible through GEO series accession number GSE184758.

## Code availability

All adapted scripts published in previous papers are available from the corresponding author on request.
