# Supplementary material for: Circadian clocks guide dendritic cells into skin lymphatics
Source: Nat Immunol. 2021 Oct 18;22(11):1375–81. doi: 10.1038/s41590-021-01040-x (PMC8553624; doi:10.1038/s41590-021-01040-x)
Supplement: Supplementary file 1 — Supplementary information on antibodies, materials and software. [file 41590_2021_1040_MOESM1_ESM.pdf]

nature portfolio

https://doi.org/10.1038/s41590-021-01040-x

Supplementary information

Circadian clocks guide dendritic cells into skin lymphatics

In the format provided by the authors and unedited

Supplementary data, materials and software

Antibodies

Antigen	Conj.	Dil.	Clone	Catalog #	Supplier
ALCAM	PE	1:100	Polyclonal	FAB1172P	R&D Systems
CCL21	Biotin	1:100	Polyclonal	BAF457	R&D Systems
CCR7 / CD197	BV786	1:25	4B12	564355	BD Biosciences
CCR7 / CD197	PE	1:24	4B12	120106	Biolegend
CD11c	PE	1:100	N418	117308	Biolegend
CD11c	APC/Cy7	1:400	N418	117323	Biolegend
CD11c	BV510	1:300	N418	117337	Biolegend
CD16/32	-	1:50	93	101302	Biolegend
CD205	PE-Cy7	1:200	205yekta	25-2051-43	Biolegend
CD301b	PerCP-Cy5.5	1:200	URA-1	146810	Biolegend
CD31/PECAM-1	APC	1:100	390	102410	Biolegend
CD31/PECAM-1	AF488	1:100	390	102413	Biolegend
CD40	BUV737	1:200	3/23	741749	BD Biosciences
CD45	PE-Dazzle 594	1:500	30-F11	103145	Biolegend
CD45	AF488	1:500	30-F11	103122	Biolegend
CD45	BV421	1:400	30-F11	563890	BD Bioscience
CD45	BUV395	1:300	30-F11	565967	Biolegend
CD80	BUV605	1:200	16-10A1	563052	BD Biosciences
CD86	BUV395	1:200	GL1	564199	BD Biosciences
CD99	PE	1:100	Polyclonal	FAB3905P	Novusbio
EPCAM/CD326	AF647	1:1000	G8.8	118212	Biolegend
EPCAM/CD326	PE	1:200	G8.8	563477	BD Biosciences
E-SELECTIN	PE	1:100	10E9.6	553751	BD Bioscience
GOLPH4/GPP130	-	1:100	Polyclonal	Ab28049	Abcam
GP38/PODOPLANIN	PE	1:100	8.1.1.	127407	Biolegend
ICAM1	PE	1:100	YNI.7.4	116108	Biolegend
JAM-A/CD321	PE	1:100	H202-106	B100-65340PE	Novusbio
JAM-A/CD321	FITC	1:200	REA854	5191028595	Miltenyi Biotech
JAM-C/CD323	APC	1:100	209628	-	Gift Dr. Beat Imhof
L1CAM	PE	1:100	555	FAB5674P	R&D Systems
LAMININ	-	1:100	Polyclonal	L9393-100UL	Sigma-Aldrich

Antigen	Conj.	Dil.	Clone	Catalog #	Supplier
LANGERIN	AF647	1:100	929F3.01	DDX0362A 647-5	Origene (Acris)
LYVE-1	AF488	1:100	ALY7	53-0443-82	eBioscience
LYVE-1	DL405	1:100	ALY7	NBP1-43411V	Novusbio
LYVE-1	-	1:100	223322	MAB2125-SP	R&D Systems
MHCII/I-A/I-E	PE/Cy7	1:1000	M5/114.15.2	107629	Biolegend
MHCII/I-A/I-E	BV650	1:2000	M5/114.15.2	107641	Biolegend
MMR1	PE	1:100	Polyclonal	FAB2535P	Novusbio
NEUROPILIN	PE	1:100	3E12	145204	Biolegend
PROX-1	APC	1:100	5G10	NBP1-30045APC	Novusbio
SEMA3A	PE	1:100	215803	IC1250P	R&D Systems
VCAM1	PE	1:100	429 (MVCAM.A)	105714	Biolegend
VE-CADHERIN	PE	1:100	BV13	NBP1-43348PE	Novusbio

Target	Host	Conj.	Dil.	Catalog #	Supplier
Rabbit	Goat	FITC	1:100	111-095-144	Jackson Immuno
Rabbit	Goat	Cy3	1:700	111-165-144	Jackson Immuno
Rat	Donkey	AF488	1:500	712-546-150	Jackson Immuno
Biotin	Streptavidin	PE	1:500	S866	Invitrogen
Biotin	Streptavidin	AF647	1:400	019-600-084	Jackson Immuno

Isotype	Conj.	Dil.	Clone	Cat. #	Supplier
Goat IgG	Biotin	1:100	Polyclonal	BAF108	R&D Systems
Goat IgG	PE	1:100	Polyconal	IC108P	Novusbio
Hamster IgG2, κ	BV605	1:200	B81-3	563012	BD Biosciences
Human IgG1	FITC	1:200	RTK2758	130-113-437	Miltenyi Biotech
Human IgG1	APC	1:200	REA-293	130-113-434	Miltenyi Biotech
Rat IgG1, κ	PE	1:100	E3-34 (RUO)	553925	BD Biosciences
Rat IgG2a	BV786	1:25	R35-95	563335	BD Biosciences
Rat IgG2a, κ	PE	1:100	RTK2758	400508	Biolegend
Rat IgG2a, κ	BUV395	1:200	R35-95	563556	BD Biosciences
Rat IgG2a, κ	BUV737	1:200	R35-95	612760	BD Biosciences
Rat IgG2a, κ	PE-Cy7	1:200	R35-95	552784	BD Biosciences
Rat IgG2a, λ	PerCP-Cy5.5	1:200	R35-95	552784	BD Biosciences
Rat IgG2b, κ	PE	1:100	RTK4530	400608	Biolegend

Target	Host	IG	Conc.	Clonality	Cat.#	Supplier
CCL21	Goat	IgG	20 µg/ml	Polyclonal	AF457	R&D Systems
Isotype control	Goat	IgG	20 µg/ml	Polyclonal	AB-108-C	R&D Systems
CD99	Rabbit	IgG	30 µg/ml	Polyclonal	Dietmar Vestweber	MPI Molecular Biomedicine
Isotype control	Rabbit	IgG	30 µg/ml	Polyclonal	AB-105-C	R&D Systems
JAM-A/CD321	Rat	IgG2b	30 µg/ml	BV1	MABT12 8	Sigma-Aldrich
Isotype control	Rat	IgG2b	30 µg/ml	141945	MAB0061	R&D Systems
JAM-C	Rat	IgG2a	30 µg/ml	H33	Gift from Beat Imhof	University of Geneva,
Isotype control	Rat	IgG2a	30 µg/ml	54447	MAB006	R&D Systems
LYVE-1	Rat	IgG2 A	30 µg/ml	22322	MAB2125 -100	R&D Systems
Isotype control	Rat	IgG2 A	30 µg/ml	54447	MAB006	R&D Systems

Chemicals, peptides, and recombinant proteins

Chemical / reagent	Catalog #	Supplier
CellTrace Violet	C34571	Thermofisher
Collagenase IV	C4-28	Sigma
CountBright™ absolute counting beads	C36950	Life Technologies
DAPI	422801	Biolegend
Dispase II	D4693	Sigma
Heparinase II & IV	H6512	Sigma
Lipopolysaccharide (LPS)	L4391	Sigma
D-Luciferine	L9504	Sigma
mCCL21	250-13	Peprotech
Tamoxifen	T5648	Sigma
Triton-X100	X100	Sigma
CellTrace Yellow	C34573	Thermofisher
R10 – RPMI 1640	10% FCS<br>20mM penicillin & Streptomycin<br>2mM L-glutamine<br>50µM β-mercaptoethanol	In house
R10 + HEPES –<br>Phenol-red free RPMI 1640	R10 + 10mM HEPES	In house

Experimental Organisms/Strains

Mouse strain	Supplier	Cat. #
Cdh5-cre/ERT2 mice	Ralf Adams, MPI Münster, Germany	N/A
Prox1-cre mice	Jackson Laboratories	#022075
Clec9a-cre mice	Barbara Schraml, LMU Munich, Germany	N/A
Cd99$^{-/-}$ mice	Dietmar Vestweber, MPI Münster, Germany	N/A
Per1$^{-/-}$*Per2*$^{-/-}$ dKO mice	Jürgen Ripperger, Urs Albrecht, University of Fribourg, Switzerland	N/A
Bmal1$^{-/-}$ *mice*	Charna Dibner, University of Geneva, Switzerland	N/A
Ccr7$^{-/-}$ mice	Cornelia Halin, ETH Zurich, Switzerland	N/A

Oligonucleotides

Primer	Sequence (5' → 3')	Supplier
Generic *Cre* F	GCG GTC TGG CAG TAA AAA CTA TC	Eurofins
Generic *Cre* R	GTG AAA CAG CAT TGC TGT CAC TT	Eurofins
Bmal1 flox F	ACT GGA AGT AAC TTT ATC AAA CTG	Eurofins
Bmal1 flox R	CTG ACC AAC TTG CTA ACA ATT A	Eurofins
Clec9acre C	AAA AGT TCC ACT TTC TGG ATG ATG A	Eurofins
Clec9acre WT	TCA CTT ACT CCT CCA TGC TGA CG	Eurofins
Clec9acre MT	GGC TCT CTC CCC AGC ATC CAC A	Eurofins
Prox1cre WT F	GTG GAA AGG AGC GTA CAC TGA	Eurofins
Prox1cre C	CAC ACA CAC ACA CGC TTG C	Eurofins
Prox1cre MT F	GCC AGA GGC CAC TTG TGT AG	Eurofins
Ccl21 F	TGAACAGACACAGCCCTCAAGA	Eurofins
Ccl21 R	CCTCTTTGCCTGTGAGTTGGA	Eurofins
Lyve1 F	TGGTGTTACTCCTCGCCTCT	Eurofins
Lyve1 R	TTCTGCGCTGACTCTACCTG	Eurofins
Cd99 F	GCGAGTGACGACTTCAACCT	Eurofins
Cd99 R	CTCCTGACGGCTTCTTGGG	Eurofins
F11r / JAM-A F	GCAGGTCAATTTGATGGACTCGT	Eurofins
F11r / JAM-A R	AGCCGGGAGGAAACTGTTGT	Eurofins
Ccr7 F	ATG TTG AGC TGC TTG CTG GTT TCG	Eurofins
Ccr7 R	TCA GTG GCT GAC CTC CTC TT	Eurofins
Lyve1 F (ChIP)	CTG GAA CTA CAG TGC TAG AGT C	Eurofins
Lyve1 R (ChIP)	TCT CTG ATG TAA ATG TGT TTG C	Eurofins
Ccr7 F (ChIP)	AGA ACT CAA TGG CCA CCC AC	Microsynth
Ccr7 R (ChIP)	TAC TAA GCC ACC TCC CCA GT	Microsynth
Ccl21 F (ChIP)	GTA TGA CTC TGT TCC AAC CTA GGT G	Microsynth
Ccl21 R (ChIP)	TGG ACC TGG TAT TTT CTT AGT TTG G	Microsynth
Per2 F (ChIP)	GCA AAT GAG GTG GCA CTC C	Microsynth
Per2 R (ChIP)	GGA GCC GCT AGT CCC AGT AG	Microsynth

Software

Software	Version	Supplier
ImageJ / Fiji	1.51n	www.imagej.net
Slidebook	6.0	3i – Intelligent Imaging Innovations
Matlab	R2018b	Mathworks
GraphPad Prism	9.1	GraphPad
Excel	2010/2017	Microsoft
Galaxy, storage platform	-	LAFUGA Gene Center
FACSDiva	8.0.1	BD
Flowjo	10.4	Flowjo, LLC
Chemotaxis and Migration Tool	2.0	Ibidi
