# Supplementary material for: Circadian clocks guide dendritic cells into skin lymphatics
Source: Nat Immunol. 2021 Oct 18;22(11):1375–81. doi: 10.1038/s41590-021-01040-x (PMC8553624; doi:10.1038/s41590-021-01040-x)
Supplement: Supplementary file 2 — Reporting Summary [file 41590_2021_1040_MOESM2_ESM.pdf]

nature research

Corresponding author(s): Christoph Scheiermann

Last updated by author(s): Aug 30, 2021

Reporting Summary

Nature Research wishes to improve the reproducibility of the work that we publish. This form provides structure for consistency and transparency in reporting. For further information on Nature Research policies, see our Editorial Policies and the Editorial Policy Checklist.

Statistics

For all statistical analyses, confirm that the following items are present in the figure legend, table legend, main text, or Methods section.

n/a	Confirmed	
☐	☒	The exact sample size (*n*) for each experimental group/condition, given as a discrete number and unit of measurement
☐	☒	A statement on whether measurements were taken from distinct samples or whether the same sample was measured repeatedly
☐	☒	The statistical test(s) used AND whether they are one- or two-sided<br>*Only common tests should be described solely by name; describe more complex techniques in the Methods section.*
☒	☐	A description of all covariates tested
☐	☒	A description of any assumptions or corrections, such as tests of normality and adjustment for multiple comparisons
☐	☒	A full description of the statistical parameters including central tendency (e.g. means) or other basic estimates (e.g. regression coefficient) AND variation (e.g. standard deviation) or associated estimates of uncertainty (e.g. confidence intervals)
☐	☒	For null hypothesis testing, the test statistic (e.g. F, t, r) with confidence intervals, effect sizes, degrees of freedom and P value noted<br>*Give P values as exact values whenever suitable.*
☒	☐	For Bayesian analysis, information on the choice of priors and Markov chain Monte Carlo settings
☐	☒	For hierarchical and complex designs, identification of the appropriate level for tests and full reporting of outcomes
☒	☐	Estimates of effect sizes (e.g. Cohen's *d*, Pearson's *r*), indicating how they were calculated

Our web collection on statistics for biologists contains articles on many of the points above.

Software and code

Policy information about availability of computer code

Data collection	Slidebook 6.0 3i – Intelligent Imaging Innovations; Galaxy, storage platform - LAFUGA Gene Center (LMU); FACSDiva 8.0.1 BD
Data analysis	ImageJ / Fiji 1.51n www.imagej.net; Matlab R2018b Mathworks; GraphPad Prism 9.1 GraphPad; Excel 2010/2017 Microsoft; Flowjo 10.4 Flowjo, LLC; Chemotaxis and Migration Tool 2.0 Ibidi

For manuscripts utilizing custom algorithms or software that are central to the research but not yet described in published literature, software must be made available to editors and reviewers. We strongly encourage code deposition in a community repository (e.g. GitHub). See the Nature Research guidelines for submitting code & software for further information.

Data

Policy information about availability of data

All manuscripts must include a data availability statement. This statement should provide the following information, where applicable:

- Accession codes, unique identifiers, or web links for publicly available datasets
- A list of figures that have associated raw data
- A description of any restrictions on data availability

All data that support the conclusions of this paper are available from the authors on request.

Field-specific reporting

Please select the one below that is the best fit for your research. If you are not sure, read the appropriate sections before making your selection.

☒ Life sciences ☐ Behavioural & social sciences ☐ Ecological, evolutionary & environmental sciences

For a reference copy of the document with all sections, see nature.com/documents/nr-reporting-summary-flat.pdf

Life sciences study design

All studies must disclose on these points even when the disclosure is negative.

Sample size	For initial experimental design, power analyses were performed to determine sample size.
Data exclusions	Data was not excluded, unless suggested via statistical testing (GraphPad, Identitication of outliers, ROUT method, Q=1%) for the real-time imaging analysis only.
Replication	All experiments were replicated at least once to prove reproducibility and only included if obtained results were the same.
Randomization	Mice were randomly allocated into different experimental groups. Human samples were obtained from patients randomly allocated to different times of the day.
Blinding	Blinding was performed for all experiments except the real-time imaging experiments.

Reporting for specific materials, systems and methods

We require information from authors about some types of materials, experimental systems and methods used in many studies. Here, indicate whether each material, system or method listed is relevant to your study. If you are not sure if a list item applies to your research, read the appropriate section before selecting a response.

Materials & experimental systems

n/a	Involved in the study	
☐	☒	Antibodies
☒	☐	Eukaryotic cell lines
☒	☐	Palaeontology and archaeology
☐	☒	Animals and other organisms
☐	☒	Human research participants
☒	☐	Clinical data
☒	☐	Dual use research of concern

Methods

n/a	Involved in the study	
☒	☐	ChIP-seq
☐	☒	Flow cytometry
☒	☐	MRI-based neuroimaging

Antibodies

Antibodies used	A complete list of antibodies including concentrations, fluorophore, clone, reference number, provider and appropriate isotype controls are found within an 'Antibody' table in the supplementary data section.
Validation	All neutralization antibodies used for DC migration were taken from publications that have validated the antibodies prior to this study, including Johnson, L. A. & Jackson, D. G. Inflammation-induced secretion of CCL21 in lymphatic endothelium is a key regulator of integrin-mediated dendritic cell transmigration. Int Immunol 22, 839-849, doi:10.1093/intimm/dxq435 (2010)); Weber, M. et al. Interstitial dendritic cell guidance by haptotactic chemokine gradients. Science 339, 328-332, doi:10.1126/science.1228456 (2013); Russo, E. et al. Intralymphatic CCL21 Promotes Tissue Egress of Dendritic Cells through Afferent Lymphatic Vessels. Cell Rep 14, 1723-1734, doi:10.1016/j.celrep.2016.01.048 (2016); Johnson, L. A. et al. Dendritic cells enter lymph vessels by hyaluronan-mediated docking to the endothelial receptor LYVE-1. Nat Immunol 18, 762-770, doi:10.1038/ni.3750 (2017); Torzicky, M. et al. Platelet endothelial cell adhesion molecule-1 (PECAM-1/CD31) and CD99 are critical in lymphatic transmigration of human dendritic cells. J Invest Dermatol 132, 1149-1157, doi:10.1038/jid.2011.420 (2012); Cera, M. R. et al. Increased DC trafficking to lymph nodes and contact hypersensitivity in junctional adhesion molecule-A-deficient mice. J Clin Invest 114, 729-738, doi:10.1172/JCI21231 (2004).

Animals and other organisms

Policy information about studies involving animals; ARRIVE guidelines recommended for reporting animal research

Laboratory animals	Male wild-type (WT) C57BL/6N mice aged 6-8 weeks were purchased from either Charles River Laboratories (Sulzfeld, Germany) or Janvier labs (Le Genest-Saint-Isle, France). Cdh5-creERT2 mice (B6) were obtained as a gift from Ralf Adams (Max-Planck-Institute for Molecular Biomedicine Münster, Germany) and crossbred with Bmal1flox/flox (B6) obtained from Jackson Laboratories to be able to target blood (BECs) and lymphatic endothelial cells (LECs). Prox1-creERT2 (B6; purchased from Jackson Laboratories, Bar Harbor,

	Maine, USA) mice were crossbred with Cdh5-creERT2-Bmal1flox/flox to obtain Prox1-creERT2-Bmal1flox/flox mice to specifically target LECs. Cdh5-creERT2 and Prox-1-creERT2 mice were given intraperitoneal tamoxifen injections for five consecutive days at the age of six weeks to induce Cre recombinase expression and excise Bmal1 at the respective flox regions. EYFP;Clec9acre mice were crossbred with Bmal1flox/flox to target conventional dendritic cells (cDCs)28. Cd99-/- mice and bone marrow (BM) cells from Ccr7 /- mice Bmal1-/- and Per2::Luc mice were used aged 6-8 weeks. Primary BM cells from Per1 /-Per2-/- mice were provided by Jürgen Ripperger and Urs Albrecht (University of Fribourg, Switzerland). All animals were housed under a 12h:12h light:dark (L:D) schedule with ad libitum access to water and food in the Core Facility Animal Models at the Biomedical Centre (LMU, Germany) or the University of Geneva (Switzerland).
Wild animals	No wild animals were used in this study.
Field-collected samples	No field collected samples were used in this study.
Ethics oversight	All animal procedures and experiments were in accordance with the ministry of animal welfare of the region of Oberbayern and with the German law of animal welfare or were approved and performed in accordance with the guidelines of the animal research committee of Geneva, Switzerland.

Note that full information on the approval of the study protocol must also be provided in the manuscript.

Human research participants

Policy information about studies involving human research participants

Population characteristics	Skin biopsies were taken from male and female adults (mean age: 74 years).
Recruitment	Skin biopsies were taken from adults presenting at the dermatosurgery unit of the Geneva University Hospitals, Switzerland, for the removal of skin tumours under local anaesthesia. Samples were taken from excessive, tumour-free, surrounding skin known as "Burow's triangles" during the reconstruction of dermatosurgical excisions. No extra incisions were made to obtain the sample and the size of the original tumour excision was not altered by this study. The sample was placed into normal saline solution immediately after it had been excised and the time of day was noted. Samples were then embedded in OCT and shock-frozen until sectioning. No potential self-selection bias or other biases were present.
Ethics oversight	Written informed consent was obtained from each individual. The sampling was conducted according to the Declaration of Helsinki and approved by the Commission Cantonale d'Ethique de la Recherche of the University Hospitals of Geneva.

Note that full information on the approval of the study protocol must also be provided in the manuscript.

Flow Cytometry

Plots

Confirm that:

- [x] The axis labels state the marker and fluorochrome used (e.g. CD4-FITC).
- [x] The axis scales are clearly visible. Include numbers along axes only for bottom left plot of group (a 'group' is an analysis of identical markers).
- [x] All plots are contour plots with outliers or pseudocolor plots.
- [x] A numerical value for number of cells or percentage (with statistics) is provided.

Methodology

Sample preparation	Crawl-out assays: Harvested and split ears were washed for 2 h and stored in R10 medium for 24 h supplemented with 1 µg/ml CCL21. Next day, ears were harvested and gently digested for 20 min at 37 °C with collagenase IV (1 mg/ml, C5138, Sigma), DNase (0.2 mg/ml, Roche) and dispase II (0.2 mg/ml, Sigma). After digestion, cells were filtered through a 70 µm cell strainer, washed and resuspended in PBS supplemented with 2% FCS and 2 mM EDTA (Sigma). Simultaneously, the medium containing the emigrated DCs was harvested and both the ear DC and medium DC populations were first Fc-receptor blocked with anti-mouse CD16/32 for 5 min at RT and subsequently stained with fluorescence-conjugated antibodies for 30 min at 4 °C. For CCR7 staining, the stain was performed separately at 37 °C prior to the other staining step. DAPI and full-bright counting beads (Thermo Fisher) were added to cells, which were then analysed by flow-cytometry using a Gallios Flow Cytometer (Beckman Coulter) equipped with a 405, 488 and 633nm laser or BD Fortessa Flow Cytometer (405, 488, 561 and 633nm lasers, BD Biosciences).<br><br>Sorting of LECs<br>To sort dermal LECs for RNA sequencing, 4 ears from two mice were pooled per biological replicate and time point. Dermal cells were isolated as stated above and prepared for sorting on a FACSAriaIIIu (BD) equipped with 4 lasers (405, 488, 561, 633 nm) in the Biomedical Centre Munich Core Facility Flow Cytometry. Live PODOPLANIN+ CD31+ LECs were sorted directly into either 350 µl TriZol-LS (Thermo Fisher, in case of RNA sequencing) or 350 µl RLT-buffer with β-ME (1:100) (Qiagen & Sigma, respectively, in case of qPCR) at 4 °C using a 100µm nozzle with a purity > 90% as determined by purity checks after every sort. Cell numbers sorted ranged between 3000 and 5000 cells / two pooled ear pairs. Directly after sorting, samples were shock-frozen on dry-ice and stored for further analysis.
Instrument	Gallios Flow Cytometer (Beckman Coulter) equipped with a 405, 488 and 633nm laser or BD Fortessa Flow Cytometer (405, 488, 561 and 633nm lasers, BD Biosciences). Sorting: FACSAriaIIIu (BD)

Software	FACSDiva 8.0.1 BD; Flowjo 10.4 Flowjo, LLC
Cell population abundance	DC: CD11c, MHCII cells (ear: 15-20%, medium: 70-80%)<br>LEC: (2-3% final gate)
Gating strategy	DC: CD11c, MHCII, EPCAM, CD103<br>LEC: Podpoplanin, CD31 (2-3% final gate)

☒ Tick this box to confirm that a figure exemplifying the gating strategy is provided in the Supplementary Information.
